# How the Extent of Protein Folding and Oligomerization Modulate Condensate Formation and Properties

**DOI:** 10.1021/acs.jpclett.5c02083

**Published:** 2025-10-21

**Authors:** Ilan Edelstein, Yaakov Levy

**Affiliations:** Department of Chemical and Structural Biology, 34976 Weizmann Institute of Science, Rehovot, 76100, Israel

## Abstract

Although proteins across the order–disorder continuum can undergo phase separation, it remains unclear how the structural states of the protein constituents influence the material properties of the resulting condensates. Here, using a coarse-grained model of a primordial peptide–RNA system, we investigate how condensates formed from ordered versus disordered peptides differ in their properties. By systematically varying the degree of foldedness and oligomerization of the peptide constituents, we find that stronger peptide–peptide interactions reduce diffusivity, whereas stronger peptide–RNA interactions destabilize the condensate. We further show that peptide conformational plasticity modulates the balance between these interactions, acting as a powerful lever for tuning the condensate properties. This work highlights how subtle changes in protein structure shape condensate architecture, dynamics, or stability and, together with experimental observations, provides a framework for understanding how the evolutionary shift from disordered to ordered peptides may have expanded the material repertoire of biomolecular condensates.

## Graphical abstract

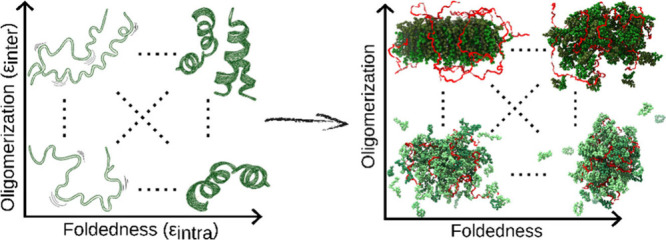

Disordered proteins are often considered main actors in liquid–liquid phase separation (LLPS), and are often deemed sufficient (and sometimes even essential) for condensate formation. Disorder is thought to facilitate multivalent, transient interactions, which are thought to promote the formation of dynamic interaction networks that spatially organize the molecules comprising the condensate.^1–3^ Disordered regions are also often enriched in specific residues or sequence motifs that are thought to act as hubs for protein–protein interactions.^4–6^ However, although protein disorder has been the primary focus of LLPS research, the extent to which protein disorder is a prerequisite for phase separation remains unclear.^7,8^

Indeed, the role played by ordered structures in modulating LLPS, although less studied, has recently attracted growing interest.^9,10^ Helical regions, in particular, appear to play a significant role, with helix–helix oligomerization emerging as a potential mechanism driving protein phase separation.^11,12^ For example, the mostly disordered C-terminal region of TAR DNA-binding protein 43 (TDP-43) contains a partially helical subregion that transiently folds into a helix upon dimerization, and tunes ribonucleoprotein granule properties^13,14^

Despite increasing evidence for the relevance of transient structural motifs in phase separation, the interplay between disordered regions in proteins and transiently folded domains remains poorly understood, especially in proteins in which both features coexist. Most experimental and computational studies have focused either on fully disordered^15–24^ or on multivalent folded proteins.^25–30^ As a result, the broad continuum between these two extremes is often overlooked, especially in the context of protein sequences that can adopt multiple conformational states. Such cases are relevant both for understanding protein maturation pathways, where a certain protein transitions from disordered to more ordered states, and for understanding evolutionary transitions from structural disorder to more stable folds. This gap in our understanding limits our ability to predict how subtle modulation of molecular structure, such as transient dimerization via a partially folded motif, affects not only phase separation but also the material properties of the condensate.

In this study, we use a coarse–grained model to investigate how the interplay between the extent of protein disorder and the protein’s propensity to dimerize modulates condensate stability and diffusivity. To explore this subject, we use the precursor–Arg (PA) peptide, a short, evolutionarily inspired sequence that phase separates in the presence of RNA (Figure 1A). PA is particularly suited for this study because it was shown that, depending on experimental conditions, it can undergo phase separation both in a prominently disordered state and in an ordered state,^31^ with the latter driven by dimerization through adoption of a transiently folded helix-hairpin-helix (HhH) motif^32^ (a fold commonly found in nucleic acid-binding proteins). By systematically varying the degrees of foldedness and oligomerization of the PA peptide, we aim to elucidate how these orthogonal features may shape the condensate properties and organization.

**Figure 1:**
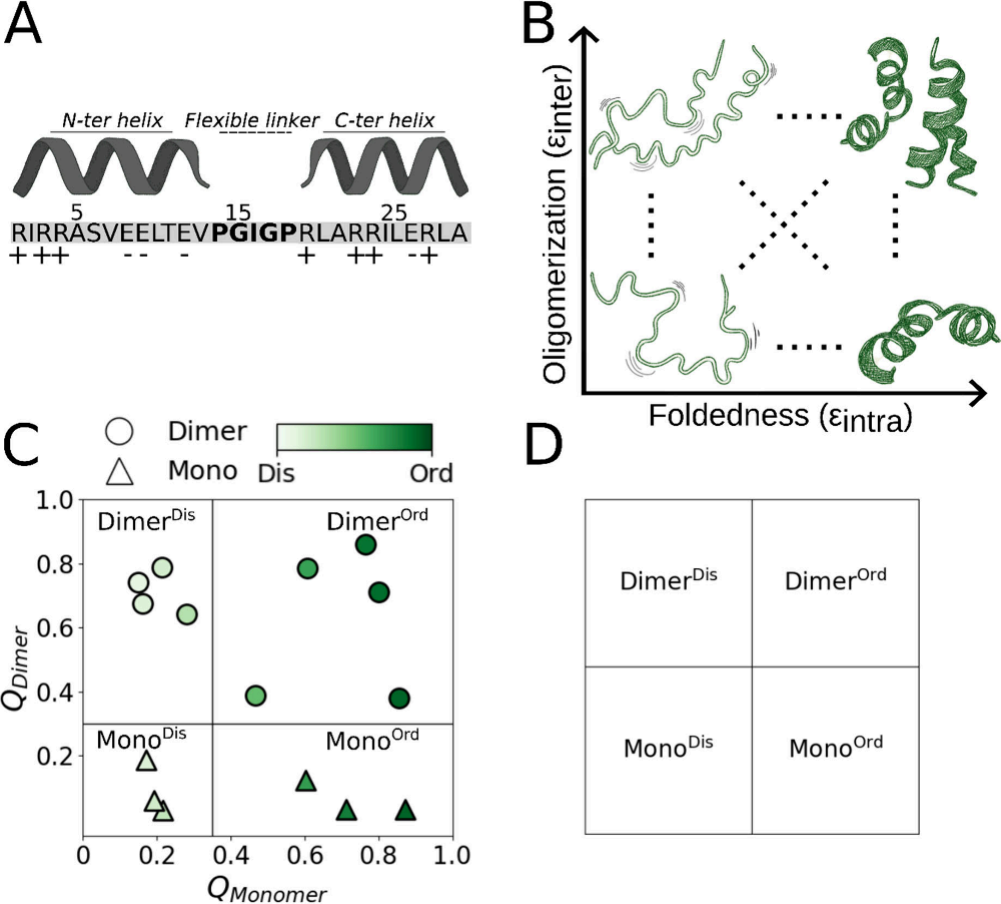
(**A**) Schematic representation of the PA peptide sequence, highlighting the N- and C-terminal regions as well as the charged residues, including arginine residues (positive) and glutamate residues (negative). (**B**) Illustration of the two axes used to generate the 15 conformational variants studied. The *y*-axis corresponds to the degree of oligomerization, ranging from a minimal interaction interface between peptide monomers (bottom) to fully dimeric states (top). The *x*-axis denotes the degree of foldedness, increasing from a mostly disordered structure (left) to a highly ordered, fully folded structure (right). The representative conformations highlight monomers and dimers as the principal oligomeric states observed across most parameter regimes (see Figure S2), despite higher-order oligomerization interfaces being in principle permitted by our model. (C) Mapping of the 15 conformational variants onto the two-dimensional conformational space defined by the averaged interfacial (*y*-axis) and intramolecular (*x*-axis) contacts measured throughout the simulations. (D) Classification of the 15 PA conformations into four groups based on the degrees to which they are oligomerized and folded: ordered monomers (mono^Ord^), disordered monomers (mono^Dis^), ordered dimers (dimer^Ord^), and disordered dimers (dimer^Dis^).

In the employed CG model, each protein residue was represented by a single bead centered at Cα, whereas RNA nucleotides were modeled with three beads for the phosphate, sugar, and base. Bonded interactions were defined via standard harmonic bond and angle potentials. Soft torsion angles were applied to 1–4 successive Cα beads (See Supporting Information Table 1).

The interaction in our model consisted of contributions of energetic terms from both transferable and nontransferable models, following the strategy of hybrid coarse-grained models used to incorporate both generic hydrophobic/electrostatic effects and structure-specific native contacts.^33–36^ Electrostatic interactions were modeled by using the Debye–Hückel potential. Hydrophobicity was modeled using the Wang-Frenkel (WF) potential with the transferable Mpipi parametrization.^37^ The structure of the PA peptide was encoded through nontransferable, specific intra– and intermolecular contact pairs (Figure S1A-B), extracted from the structure of a previously reported dimeric conformation.^32^

The specific contact pairs, were identified via the shadow algorithm,^38^ and modeled using a 12–10 Lennard-Jones potential of the form $V\left(r\right)=\epsilon_{inter/intra}\left(5{\left(\frac{\sigma }{r}\right)}^{12}-6{\left(\frac{\sigma }{r}\right)}^{10}\right)$ . To gradually decrease the degree of foldedness, we tuned the depth of the energy minima for the intramolecular contacts (ϵ_*intra*_). Similarly, to decrease the propensity of the protein monomers to participate in dimeric interactions, we tuned the strength of the intermolecular contacts (ϵ_*inter*_). In all systems, the specific intermolecular contact pairs were extended to any pair of monomers present in the system, thus allowing monomers that were initially associated and became separated to re–dimerize fully or partially with any other available monomer (Figure S1).

In order to probe multiple transiently folded conformations of the PA peptide on the continuum between the fully folded dimeric form and the completely disordered monomeric form, we investigated 15 variants, each characterized by a different combination of ϵ_*intra*_ and ϵ_*inter*_ values, where ϵ_*intra*_ = 1, 2.5, or 4 and ϵ_*inter*_ = ∼ 0, 1, 2, 3, or 4.

Since in our model each peptide is allowed to partially or fully reform a dimeric interface with any other peptide present in the simulation, the formation of higher-order oligomers is in principle possible. We therefore investigated the preferred oligomeric state of each of the 15 simulated systems by computing the mean number of oligomeric partners. This was done by evaluating the strength of the intermolecular interactions between every peptide in the condensate throughout the simulation. Peptides were then classified as interacting or noninteracting based on an energy cutoff (Figure S2A). Overall, the mean number of oligomeric partners ranged from 0 to 1 across most systems with the notable exception of those with both high ϵ_*inter*_ and low ϵ_*intra*_ parameters. In these four cases, extended conformations coupled with strong intermolecular interactions enabled the formation of partial higher-order oligomers (Figure S2B). For the remaining systems, increases in both ϵ_*inter*_ and ϵ_*intra*_ correlated with a preferred dimeric state (∼1 oligomeric partner per peptide), whereas reductions in these parameters correlated with a monomeric state (∼0 partners). Only two systems fell between these extremes, namely, ϵ_*intra*_ = 2.5, ϵ_*inter*_2 and ϵ_*intra*_ = 4, ϵ_*inter*_ = 1, which showed intermediate mean values of ∼ 0.4 and ∼ 0.2 oligomeric partners, respectively (Figure S2B).

We quantified the degree of protein foldedness and the degree of dimerization in each variant using two parameters: *Q*_*Monomer*_ and *Q*_*Dimer*_. The *Q*_*Monomer*_ parameter measures the fraction of intramolecular interactions that define a folded monomeric PA, and ranges in value from 0 (fully unfolded, *i.e*., maximally disordered) to 1 (fully folded, i.e., maximally ordered), whereas the *Q*_*Dimer*_ parameter measures the fraction of intermolecular interactions that define the dimeric interface of PA, with its values ranging from 0 (entirely monomeric) to 1 (entirely dimeric).

We quantified the degree of orientational alignment of peptides parallel to each other along a common axis within each condensate by computing the nematic order parameter.^39^ For a system of N peptides with orientation unit vectors *e*, *Q* the ordering matrix was defined as $Q=\frac{1}{2N}\overset{N}{\underset{i=1}{\sum }}3e_{\alpha }^{i}e_{\beta }^{i}-\delta_{\alpha \beta }$ where α, β ∈{*x*, *y*, *z*}. Diagonalization of *Q* yields its eigenvalues, and the nematic order parameter S was then defined as the largest eigenvalue of *Q*.

All simulations were conducted in OpenMM^40^ and run in a 30 nm cubic box with periodic boundaries that contained (unless stated otherwise) 120 peptides and four 100-nt polyU molecules. Following energy minimization and an equilibration phase sufficient to allow condensate formation (Figure S10), a 1 μs production run was performed with a 10 fs time step. Three independent simulations were performed for each system, yielding a total aggregate simulation time of 3 μs. Further details regarding the model, simulation setup, phase diagrams, calculation of the diffusion coefficient, and structural and energetical analysis are provided in the Supporting Information.

To study how the degree of protein foldedness and the degree of oligomerization influence the properties of protein condensates, we designed a set of 15 PA peptide variants that differed in terms of both monomer folding (*i.e*., degree of foldedness, quantified by *Q*_*Monomer*_) and oligomerization propensity (*i.e*., degree of dimerization, quantified by *Q*_*Dimer*_). The 15 designed PA systems span a continuum from fully folded, dimeric PA (*i.e*., *Q*_*Monomer*_ = *Q*_*Dimer*_ = 1) to fully disordered, monomeric PA (*i.e*., *Q*_*Monomer*_ = *Q*_*Dimer*_ = 0), enabling a systematic investigation of how gradual changes along the foldedness and oligomerization axes affect the properties of PA condensates (Figure 1B).

Although our model allows in principle each peptide to interact with multiple peptides, *Q*_*Dimer*_ as well as the intermolecular interaction energy cutoff analyses indicate that the preferred oligomeric states emerging within the condensed phase of most system is predominantly either monomeric or dimeric states (Figure S2). Accordingly, we first classified the 15 PA systems into four groups: folded (i.e., highly ordered) monomers (Mono^Ord^), disordered monomers (Mono^Dis^), folded dimers (Dimer^Ord^), and disordered dimer (Dimer^Dis^) (see Figure 1D). A PA is classified as ordered (i.e., folded) if it satisfies *Q*_*Monomer*_ > 0.35 when simulated in the context of the condensate. Similarly, a PA is classified as dimeric if it satisfies *Q*_*Dimer*_ > 0.3 when simulated in the condensate. It should be noted that due to the gradual approach adopted in this work, some systems fall near the midpoint of the range of *Q*_*Dimer*_ and *Q*_*Monomer*_ parameters, particularly the two systems at *Q*_*Dimer*_ = ∼ 0.4. To avoid classification threshold bias, we tested a stricter criterion of *Q*_*Dimer*_ > 0.5. This reclassified the two intermediate systems as monomers rather than dimers, without altering the observed trends (Figure S14).

Following these definitions, the 15 designed PA systems comprise six monomeric and nine dimeric variants of PA. The monomeric PA variants include three disordered and three ordered variants. The dimeric PA variants include four disordered and five ordered dimeric PAs. The degree of monomer foldedness (which is correlated with the proportion of monomers adopting a helical conformation) (Figure S8B) is visually represented throughout this work using a green color gradient. Lighter tones correspond to low helical content, indicating predominantly disordered conformations within the condensate, whereas darker tones reflect a higher helical content within the condensate, indicating predominantly ordered conformations (Figure 1C).

To construct the phase diagram of each of the 15 PA variants, each variant was simulated in the presence of RNA molecules across a range of temperatures (Figure 2A-B). For consistency and to allow comparison between condensates with comparable properties, all systems were analyzed at different absolute temperatures but at a similar relative stability (*T*/*T*_*C*_ ≈ 0.9). This regime was chosen because all systems exhibit phase separation under these conditions (Figure S3).

**Figure 2:**
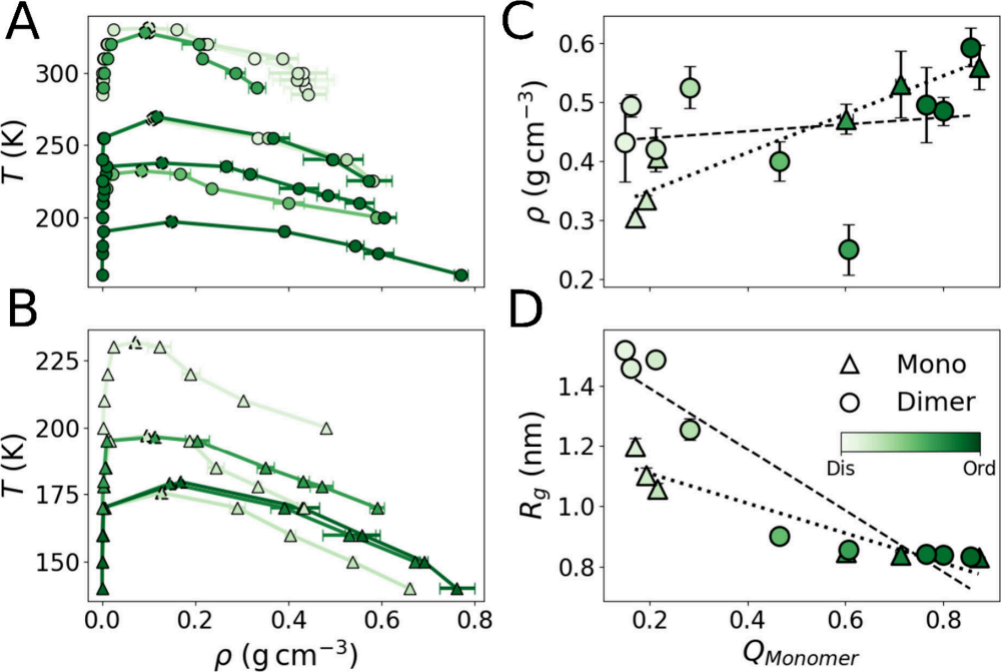
Phase diagrams for the (A) dimeric and (B) monomeric systems. Each curve is colored according to the degree of foldedness at T/*T*_c_ = 0.9, with darker tones indicating higher foldedness (i.e., more monomers adopting a highly ordered folded conformation). The critical temperature obtained from the fitting for each system is indicated by dashed markers. (C) Condensate averaged density plotted against the fraction of intramolecular interactions, with the linear fits showing positive correlations for both monomers (dashed line) and dimers (dotted line). (D) Radius of gyration (*R*_*g*_) of peptides within the condensate plotted against the fraction of intramolecular interactions, with the linear fits showing negative correlations. Larger *R*_*g*_ values correspond to extended conformations in disordered systems. In all panels, darker tones represent higher foldedness (greater helical content), whereas lighter tones indicate decreased foldedness. Values represent the mean of replicate averages, and error bars denote the standard deviation of these averages.

Interestingly, we found that increases in foldedness (or in *Q*_*Mono*_) correlate positively with condensate density, with a maximal density increase of ∼ 50% when comparing disordered (lighter green) monomers with ordered (darker green) monomers (see Figure 2C), indicating a link between condensate density and intramolecular interactions. In contrast, increase in *Q*_*Dimer*_ (a proxy for oligomerization propensity) does not necessarily result in increase in condensate density (Figure S9A), as might be expected if condensate density was simply a byproduct of systems with more favorable specific intermolecular interactions. This suggests that condensates formed by more disordered constituents are less efficiently packed than those formed by more ordered ones. A likely explanation is that disordered PA peptides adopt, on average, more extended conformations within the condensate, as indicated by their larger radii of gyration (*R*_*g*_) (Figure 2D), which may reduce their packing efficiency. Nonetheless, the relationship among the degree of foldedness, *R*_*g*_, and condensate packing seems to be more complex. For example, disordered dimeric PA variants (Figure 2D, light green circles) exhibit large *R*_*g*_ values yet form a tightly packed condensate likely due to a unique mode of interaction with RNA, as discussed below. Moreover, RNA conformations themselves are influenced by condensate density, with the average *R*_*g*_ of RNA molecules decreasing in denser condensates (Figure S9C). Both monomerization and folding, which were shown to enhance peptide–RNA interactions (Figure 3), were also found to promote more compact RNA conformations inside of the condensate (Figure S9C).

**Figure 3:**
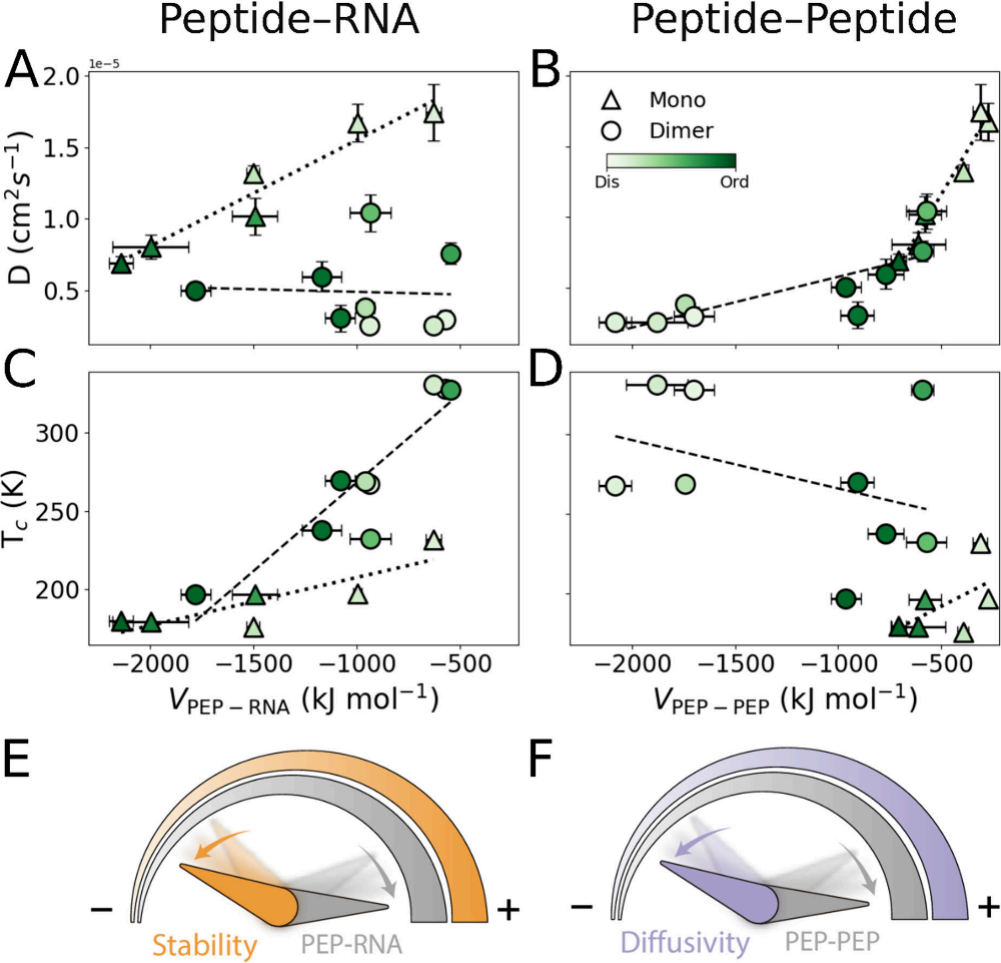
Average diffusion coefficient (D) of peptides within the condensate, plotted as a function of the mean interaction energy for (A) peptide–RNA interactions and (B) peptide–peptide interactions. Critical temperature (*T*_*c*_) of phase separation is plotted as a function of the mean interaction energy for (C) peptides–RNA interactions and (D) peptide–peptide interactions. (E) A schematic illustration linking the strength of peptide-RNA intermolecular interactions to the condensate stability. (F) Schematic illustration linking the strength of peptide–peptide intermolecular interactions to the peptide diffusion within the condensate. In (E) and (F), an increase in interaction strength or in condensate property is denoted by “+”, while a decrease is denoted by “–”. In all plots, darker green color indicates higher degrees of foldedness. Values represent the mean of replicate averages, and error bars denote the standard deviation of these averages.

To investigate how oligomerization and foldedness influence condensate properties, we calculated the critical temperature (*T*_*c*_) and the average peptide diffusivity within the condensate (i.e., diffusion coefficient D) for each of the 15 simulated PA variants. These properties were then correlated with the average peptide-RNA and peptide–peptide interaction energies (*V*_*PEP*–*RNA*_ and *V*_*PEP*–*PEP*_, respectively) measured within the condensate. In both cases, interaction energies were computed as the sum of electrostatic and hydrophobic contributions while excluding intramolecular contributions (i.e., internal interactions within a peptide or an RNA). Thus, *V*_*PEP*–*PEP*_ includes only peptide–peptide interactions, and *V*_*PEP*–*RNA*_ only peptide–RNA interactions. The relationship between intra- and intermolecular peptide interaction energies is provided in the Supporting Information (Figure S9B).

Protein diffusion within the condensates was found to follow a normal diffusivity regime, as indicated by the diffusion exponent (α) of ∼ 1 (Figure S5A), and to depend on the preferred oligomeric state of the system. Monomeric variants of the PA peptides were found to exhibit higher diffusivities within the condensate compared to their dimeric counterparts, as indicated by their upward shift in diffusion coefficient values (Figure 3C–D) (Figure 3A–B). This difference in diffusion coefficients can be traced to distinct intermolecular interactions profiles, in which peptide–peptide and peptide–RNA interactions exert opposing effects on diffusivity. In particular, monomeric PA variants tend to interact more strongly with RNA than dimeric variants, as evidenced by the leftward shift in their measured averaged peptide–RNA energies (Figure 3A), while simultaneously forming weaker peptide–peptide interactions, as indicated by a rightward shift in their measured peptide–peptide interaction energies (Figure 3B). Dimeric PA variants, on the other hand, display the exact opposite trend, tending to interact more strongly with other peptides (Figure 3B), but more weakly with RNA (Figure 3A). These opposing trends suggest that more favorable peptide–peptide interactions are consistently associated with reduced diffusivity within the condensate (Figure 3B), and that the oligomeric state of the system affects the diffusivity by balancing intermolecular versus intramolecular interactions.

Looking at the effect of foldedness on diffusivity, we found that increased foldedness correlates strongly with decreased diffusivity for the monomeric PA variants (*R* ^2^ = 0.94), whereas it shows only a weak positive correlation with diffusivity in dimeric variants (*R* ^2^ = 0.17) (Figure 3A-B and Figure S8F). These differences reflect the distinct ways in which foldedness shifts the balance of intermolecular preferences toward peptide–peptide versus peptide–RNA interactions. Systems with a greater degree of foldedness interact more strongly with RNA, as evidenced by the leftward shift in their measured peptide–RNA interaction energies (Figure 3A, darker green shades). Indeed, peptide–RNA interactions are negatively correlated with foldedness, with *R* ^2^ values of ∼ 0.8 for monomeric and ∼0.4 for dimeric PA variants (Figure S8C). Peptide–peptide interactions energies differently correlate with foldedness for monomeric and dimeric PA. Monomeric PA variants show a strong negative correlation (*R* ^2^ = 0.96), whereas dimeric variants show a positive correlation (*R* ^2^ ≈ 0.7) (Figure S8D). Consequently, decreased foldedness results in tighter peptide–peptide interactions for dimeric PA but weaker peptide–peptide interactions for monomeric PA.

Together, these findings indicate that foldedness and oligomerization differentially tune the balance of interactions within the condensate. Foldedness and monomerization bias the system toward peptide–RNA interactions, whereas dimerization shifts the balance toward peptide–peptide interactions. Moreover, diffusivity is consistently negatively correlated with peptide–peptide interactions (Figure 3F), underscoring how a shift in one specific intermolecular interaction can directly reshape the diffusive properties of the condensates.

RNA diffusion within the condensates followed an anomalous regime, with α values ranging between ∼ 0.5–0.6 (Figure S5B), consistent with subdiffusive behavior and likely arising from interactions with peptides within the condensate. Systems in which PA variants preferred monomeric conformations exhibited slightly lower α values (∼0.5) compared to systems with preferred dimeric conformations (∼0.6) (Figure S5B). Notably, the folded monomeric PA variants also tend to interact more strongly with RNA than disordered monomeric PA (Figure 3A) and consequently RNA *R*_*g*_ is smaller (Figure S9C). These observations suggest that stronger peptide–RNA interactions promote more compact RNA conformations, likely because the electrostatic repulsion between the RNA molecules is more effectively screened, thereby slightly reducing the degree of RNA subdiffusion (slightly raising α).

Regarding condensate stability, as estimated by *T*_*c*_, dimeric conformations were found to exhibit greater condensate stability, compared to their monomeric counterparts, as indicated by their upward shift in *T*_*c*_ values (Figure 3C–D). As with diffusivity, this difference can be explained by the distinct contributions of peptide–peptide and peptide–RNA interactions. In particular, stronger peptide–RNA interactions are correlated with decreased condensate stability in both monomeric and dimeric PA variants (Figure 3C). The relationship between stability and peptide–peptide interactions is more complex. Stronger peptide–peptide interactions are correlated with decreased stability in monomeric PA variants but improved stability in dimeric variants (Figure 3D).

Condensate stability is also affected, albeit more subtly, by the degree of peptide foldedness. An increase in foldedness tends to be weakly correlated with reduced condensate stability (Figures 3C–D) with a *R* ^2^ *of* ∼ 0.3 for both monomeric and dimeric PA variants (Figure S8E). Figure 4 shows representative structures of the condensate for each of the four categories of PA states. These snapshots illustrate the interaction between different PA peptides and their organization around the RNA.

**Figure 4:**
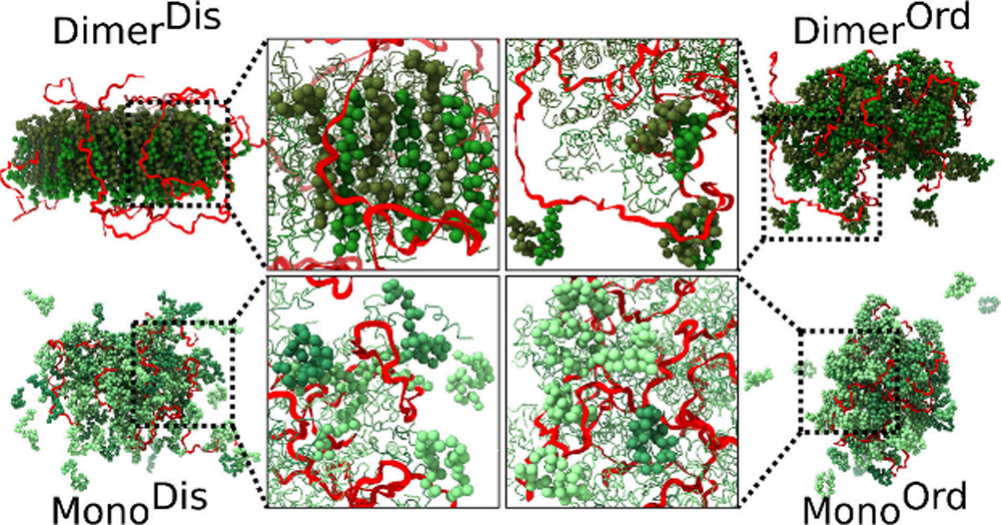
Representative snapshots of the condensate formed by dimers (upper panel) and monomers (lower panel). Foldedness increases from left to right, showing condensates formed by disordered peptides (left panels) and ordered peptides (right panels). RNA is represented in red, dimers are represented in two shades of greens, and monomers are represented in a spectrum of green shades.

Overall, this analysis reveals how peptide–peptide and peptide–RNA interactions each regulate distinct condensate properties (Figure 3E–F). More favorable peptide–peptide interactions are linked to reduced peptide mobility within the condensate (Figure 3A–B and 3F). While in contrast, peptide–RNA interactions are associated with lower *T*_*c*_ values (Figure 3C–D and 3E). Notably, diffusivity and stability tend to behave differently for different preferred oligomeric states. Systems with preferred monomeric conformations tend to exhibit higher diffusivity but lower stability, whereas systems with preferred dimeric conformations tend to display lower diffusivity alongside greater stability (Figure 3). This suggests that the balance between the two molecular interactions is tuned by the structural characteristics of the system (*i.e*., oligomeric state and degree of foldedness). Systems with a higher degree of foldedness and systems with preferred monomeric conformations shifted the balance toward peptide–RNA interactions, while systems with preferred dimeric conformations shifted toward peptide–peptide interactions. This illustrates how structural changes can reshape how similar residue-level hydrophobic and electrostatic interactions are displayed in intermolecular interactions, ultimately resulting in distinct properties of the condensates.

Of the four condensate groups defined above, Dimer^Dis^ exhibits a particularly distinct behavior. Disordered dimeric PA peptides form the least diffusive and most stable condensates (Figure 3A–B), while also forming condensates with a higher-than-expected density (Figure 2C). They also undergo the largest increase in *R*_*g*_ upon entering the condensate (Figure S9D). These characteristics, which are also reflected in the unique structural organization of the Dimer^Dis^ group (see Figure 4, top left corner), prompted us to investigate what sets Dimer^Dis^ apart from the other groups. Notably, peptides in the Dimer^Dis^ group transition from an isotropic phase (in which the same peptide properties are obtained from all directions of measurement) to a nematic phase (in which the long axes of the peptides tend to align parallel to each other along a common axis), as revealed by the average value of the nematic order parameter (S ≈ 0.6), which is substantially higher than the value (S ≈ 0.4) observed for all other groups (see Figure 5A). Inside this nematic phase, the peptides self-assemble into elongated and highly ordered structures, in contrast to their disordered monomer counterparts (Mono^Dis^; Figure 4, bottom left corner). Structural analysis suggests that RNA primarily engages in electrostatic interactions with the positively charged N-terminal regions of the peptides (Figure 4). This results in a preferred orientation, with N-termini facing outward toward the RNA-rich interface and C-termini pointing inward (Figure 5A, right panel). Removing RNA from the simulations resulted in a decrease in nematic order for these PA variants (Figure S10), further highlighting the role of RNA in stabilizing the nematic phase. Given the outlier behavior of this group, we assessed whether their inclusion biased the observed correlations. Excluding them did not affect the key relationships reported above (Figure S13)­.

**Figure 5:**
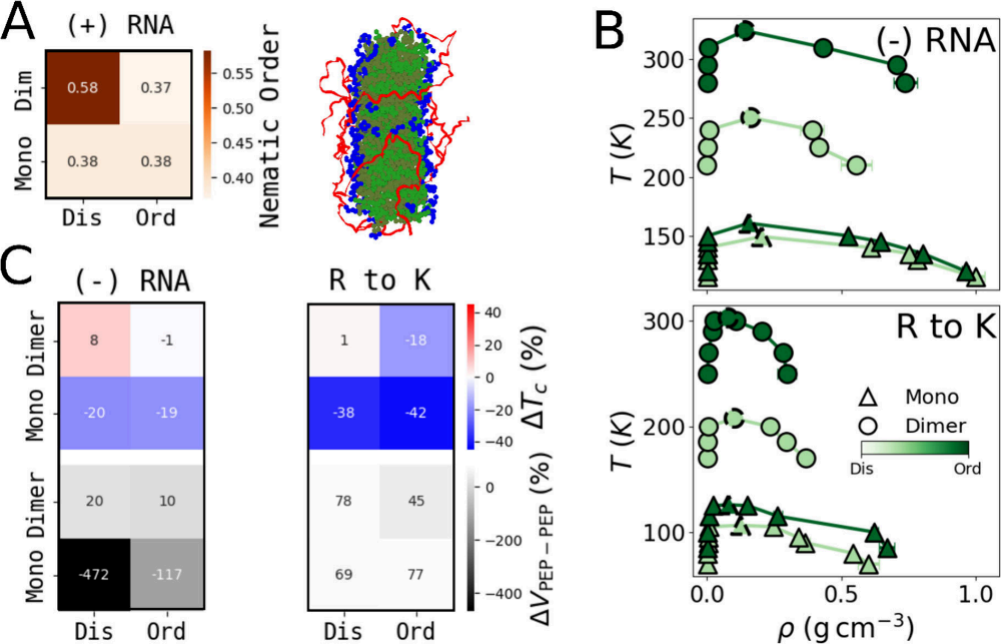
(A) Averaged nematic order parameters for each group of protein condensates in the presence of RNA (left). Conformation showing the nematic structure formed by the dimer^Dis^ group, with RNA molecules represented in red, the positively charged N-termini of the PA peptides shown in blue, and the remaining peptide dimers shown in shades of green (right). (**B**) Phase diagrams in the absence of RNA (top) and with all arginine residues mutated to lysine residues (R to K) (bottom). The critical temperature is indicated by dashed markers. (**C**) Change in stability (as measured by T_C_, top panels) and peptide–peptide interactions (Δ*V*_*PEP*–*PEP*_, bottom panels), represented by the relative error between the mean *T*_*c*_ and *V*_*PEP*–*PEP*_ of each representative condensate group. Shown in the absence of RNA (left) and following the R to K substitutions (right).

Having shown that diffusivity within PA condensates and their stability are modulated by the strength of peptide–peptide and peptide–RNA interactions, we sought to investigate how perturbing these intermolecular interactions affects phase separation and condensate properties. To do so, we focused on four representative PA systems, one from each subtype group, and simulated them in the absence of RNA or after mutating all arginine residues to lysines (R to K). Lysine was chosen to maintain the overall charge of the peptide, while reducing hydrophobic interactions. Each representative system was then simulated across a range of temperatures to construct a phase diagram and determine its *T*_*c*_ (Figure 5B). Systems were compared at the same relative stability (*T*/*T*_*C*_ ≈ 0.9), since under these conditions all systems exhibited phase separation (Figure S4).

Consistent with previous observations for PA condensates,^32,41^ we showed that RNA overall enhances PA condensate formation, as evidenced by reduced stability upon RNA removal (Figure 5C). Condensate stability is more affected by the removal of RNA when the condensate is formed by monomeric rather than dimeric PA (Figure 5C). Both disordered and ordered monomeric PA show a decrease of approximately 20% in their respective *T*_*c*_ values upon RNA removal (Figure 5C). Interestingly, in the presence of RNA, monomeric PAs generally interact with RNA more favorably (Figure 3). This seems to indicate that while stronger interactions with RNA tend to generally have a negative impact on stability of the condensate (Figure 3C), the observed decrease in stability upon RNA removal suggests that RNA nonetheless plays a crucial role at the molecular scale in promoting condensate formation by monomers. A possible explanation is that RNA serves to screen repulsive electrostatic forces thereby serving as a scaffold that enhances peptide connectivity. Supporting this view, we showed that systems with preferred monomeric conformations exhibit more compact RNA conformations (Figure S9C) and argued that peptide–RNA interactions screen electrostatic repulsion within the RNA molecules, thereby allowing their ends to approach one another. Therefore, RNA may have dual effects on peptide interactions, on the one hand, destabilizing condensates by competing with peptide–peptide interactions, while on the other hand, promoting peptide connectivity through electrostatic screening, as supported by the decrease in peptide–peptide interactions upon RNA removal (Figure 5C), and increase in interactions with increasing RNA length in isothermal conditions (Figure S12). This view is conceptually consistent with recent study showing that RNA can act either as a surfactant-like destabilizer or as a scaffold-like stabilizer of condensates, depending on its length.^42^ In our studied system, the competitive effect dominates, leading to an overall reduction in condensate stability. Upon RNA removal, however, the scaffolding contribution is also lost; thus, although competition is relieved, so too is RNA’s ability to promote peptide connectivity. The net effect is a reduction in stability, which is especially pronounced for monomeric variants that possess a lower baseline propensity for peptide–peptide interaction (Figure S12), making them more dependent on RNA than the dimeric variants.

The R to K substitutions also exert a strong effect on the condensate properties, as evidenced by the decrease in stability (Figure 5C). While the substitutions conserve the electrostatic contribution of intermolecular interactions, it weakens the hydrophobic contribution. The reduction in stability is more pronounced for monomers (∼40% decrease in *T*_*c*_) than for dimers (∼20% decrease in the ordered variant and negligible effect in the disordered variant). Interestingly, this reduction in stability also coincides with an increase in the number of peptide–peptide interactions (Figure 5C). This can likely be explained by the reduced ability of lysine, compared to arginine, to interact with RNA, consistent with previous work showing that arginine binds polyU more strongly than lysine in biomolecular condensate.^43^ A weaker propensity for peptide–RNA interactions is expected to reduce competition for both peptide binding and RNA-mediated screening and scaffolding. The reduced peptide–RNA interactions also increase the availability of the peptide for self-interaction, accounting for the observed rise in peptide–peptide interactions.

By contrast, Dimer^Dis^ showed increases in peptide–peptide interactions, and only minor changes in stability for both perturbations (namely, without RNA and following the mutation of R to K residues) (Figure 5C). Notably, this group was also unique in displaying nematic behavior (Figure 5A). In this system, RNA is located mostly outside of the assembly and interacts via electrostatic interactions with the positively charged N-terminal regions of the outward-pointing peptides. Because the two chains in each dimer form strong dimeric interface, the dimers adopt an elongated, rod-like shape with heightened rigidity, consistent with the high *R*_*g*_ values observed (Figure 2D). In the absence of RNA, this ordering becomes less favorable, as indicated by a reduced nematic order parameter (Figure S10), suggesting that removal of RNA results in increase in peptide–peptide interactions (Figure 5C) due to the weaker ordering of peptides. In the case of the R to K mutation, RNA is still present and capable of concentrating peptides, though less effectively than with arginine residues. The substitution increases the probability for peptide self-interaction, producing a stronger rise in peptide–peptide interactions than that observed upon RNA removal (Figure 5C).

Erwin Schrödinger famously described two fundamental ways of producing order, ‘order from disorder’ and ‘order from order’.^44^ The former arises from statistical principles, where predictable macroscopic behavior emerges from the collective stochastic motion of large numbers of atoms. The latter refers to systems in which each component is precisely arranged, by engineering design or evolution, to sustain a specific function. In a similar manner, phase separation of a biomolecular condensate, in itself a form of emergent order, can be driven by both disordered and ordered molecular constituents. Proteins, as the primary constituents of most biomolecular condensates, are often classified dichotomously as either adopting a disordered or ordered state.^45^ Although disordered states lack a stable three–dimensional structure and remain highly flexible, sampling a heterogeneous ensemble of flexible conformations, they are believed to promote multiple transient, weak multivalent interactions that act as a driver for phase separation.^46,47^ In contrast, ordered states adopt well-defined structures and interact through precisely positioned intermolecular interfaces. However, the extent to which condensates assembled from ordered versus disordered constituents exhibit fundamentally distinct properties remains an open question. Addressing this question requires a model system in which the degree of protein structure can be systematically tuned while maintaining the ability of the proteins to undergo phase separation.

Here, we addressed this question by using the PA peptide as a model system. This peptide was chosen because it has been previously shown experimentally to reversibly form α–helical dimers and undergo phase separation in the presence of RNA.^32^ Moreover, it was previously shown in a study combining both EPR spectroscopy and all-atom simulations that even a heterochiral variant of the PA peptide (composed of alternating D- and L-amino acids) remains capable of both partial dimerization and RNA-driven phase separation.^41^ Atomistic simulations suggested important differences at the molecular scale in terms of folding and oligomerization propensities between the homochiral and heterochiral variants; nonetheless, the extent to which variations along these structural axes influence condensate properties remained unresolved. Experimentally probing such subtle modulation remains highly challenging, making computational approaches uniquely valuable.^48^ Coarse-grained molecular dynamics, in particular, can capture the essential physical interactions that drive phase separation while enabling the efficient sampling of large systems over extended time scales required for resolving emergent condensate properties. Therefore, to address this gap, we developed a hybrid coarse-grained model, informed by these experimental observations, that combines nonspecific contributions (hydrophobic and electrostatic interactions) with system-specific intra- and intermolecular interactions. Within this framework, we were able to independently tune two parameters that control the degree of peptide foldedness and the degree of oligomerization while keeping the residue-level hydrophobicity and electrostatic interactions unchanged. This approach allowed us to simulate condensate formation across the full parameter space, encompassing all combinations of states from complete disorder to full helicity and from purely monomeric to entirely dimeric peptides.

Several experimental studies have demonstrated that variations at the molecular scale can influence condensate properties.^49–51^ For example, in the case of superoxide dismutase 1 (SOD1), condensation biases an immature form of SOD1 toward unfolded states that are susceptible to aggregation, while a more mature form of the protein is much less affected.^52^ In the case of the PA, both folded and disordered variants were capable of undergoing phase separation in the presence of RNA, despite notable differences in their critical temperatures (Figure A-B). Furthermore, we observed that foldedness modulates the balance of intermolecular interactions within the condensate, on one hand, tending to increase the ability of peptides to interact with RNA, and on the other hand, constraining peptide–peptide interactions to a narrower interaction interface. These differences in molecular interactions preferences translate into different condensate properties such as convergence in diffusivity and density (Figure 3B and 2C) and decrease in stability (Figures 3C).

Reciprocally, several studies have demonstrated that the formation of condensates can actively reshape the foldedness of the protein constituents. For example, helical conformations in condensates formed by poly–lysine peptides,^53^ increased helicity inside droplets of human serum albumin protein,^54^ and extended or β-like structures induced by RNA interactions in the SARS-CoV-2 nucleocapsid protein.^55^ Similarly, higher–order assemblies rich in β-sheet content have been observed in FUS condensates driven by RNA binding.^56^ Protein-RNA interactions within condensate was also shown to regulate phase separation and influence protein structure, for instance RNA was shown to not only regulates condensate formation through electrostatic interactions with arginine-rich motifs,^57^ through sequence-dependent base stacking and pairing interactions,^58^ or through chemically specific interactions with diverse amino acid residues that govern the intricate interplay of peptide–RNA and peptide–peptide interactions,^59,60^ but to also modulates the conformational dynamics of proteins upon binding.^61,62^ Taken together, this suggests that the condensate microenvironment, and particularly RNA–mediated interactions, can shift protein foldedness along the disordered–ordered continuum. Therefore, if folding can shape condensate properties, and condensates can in turn reshape folding, then specific sequences may have evolved to fine–tune their condensate microenvironment and hereby produce condensates with distinct material properties, tailored for specific functions.

Previous work has emphasized the central role of disorder in LLPS across diverse protein systems of varying complexity, including FUS, hnRNPA1, and DDX4, which phase separate via multivalent, low-affinity interactions often modulated by RNA binding.^63–65^ Growing evidence indicates that folded structures can also drive phase separation, either through oligomerization via discrete interfaces^12,66^ or via conformational rearrangements that expose interaction surfaces that promote multimerization upon phase separation.^67^ Additionally, folded domains, such as the folded RNA recognition motifs of hnRNPA1, can modulate the salt dependence of the hnRNPA1 phase behavior through their interactions with disordered regions. Specifically, interdomain interactions enhance hnRNPA1 phase separation under low salt conditions whereas screening of such of interactions under high salt conditions increases protein solubility and abolishes phase separation.^68^ Even predominantly disordered proteins might contain subregions with transiently folded alpha-helices,^69^ which are capable of acting as multivalent ‘hotspots’.^70^ For instance, Efg1, which is an important fungal transcription factor, was shown to act as a hub for both protein–protein and protein–RNA interactions.^71^ Moreover, a partially helical region in the disorder C-terminal domain of TDP43 was shown to mediate TDP43 phase separation by acting as an essential multivalent hub for dimerization or higher order oligomerization,^13,14,72^ whereas phosphomimetic substitutions in both the transiently helical region of the C-terminal^73^ and in the highly conserved folded N-terminal domain of TDP43 suppress or decrease phase separation.^74^ Interestingly, increasing partial helicity does not always result in an increased phase separation propensity. In the fungal RNA–binding protein Whi3, formation of a transient alpha–helix prohibits dimerization and therefore phase separation.^75^ Taken together, these findings highlight oligomerization as an increasingly recognized driver of LLPS, distinct from the typical disordered protein driver. Whereas disorder-driven LLPS emerges from numerous weak and transient interactions, oligomerization relies on precise interaction interfaces.

Our results suggest that the oligomeric state of the protein or peptide constituents influences condensate properties, by tuning the balance between peptide–peptide and peptide–RNA interactions. Indeed, condensates composed of monomeric PA variants were shown to preferentially engage with RNA, whereas dimeric variants favor interactions with neighboring peptides. These distinct preferences map onto different condensate properties, where stronger peptide–peptide interactions reduce diffusivity within the condensate, while stronger peptide–RNA interactions are associated with reduced stability. As a result, monomeric systems tend to form condensates that are more dynamic but less stable, whereas dimeric systems produce more stable but less dynamic condensates. These differences also intersect with the dual role of RNA in shaping the condensate stability. Since predominantly dimeric PA systems intrinsically possess strong peptide–peptide networks, the main role of RNA in those systems is competitive, leading to a largely monotonic dependence of condensate stability on RNA concentration. By contrast, monomeric systems, with weaker intrinsic connectivity, are expected to display a nonmonotonic trend, where RNA can initially favor peptide–peptide networks, but at higher concentrations this effect reverses as competition for peptide binding dominates. This echoes the experimental demonstration of RNA-modulated re-entrant phase behavior^76^ and may reflect a broader feature of systems governed by electrostatics.^77^ It is also consistent with experimental phase diagrams of the PA peptide where the predominantly dimeric homochiral form is broadly stable while the weakly dimeric heterochiral form shows a narrow, bell-shaped stability profile upon increasing RNA concentration.^41^ Together, these observations suggest that oligomerization redirects the balance of molecular interactions in ways that selectively modulate the condensate properties. More broadly, this illustrates how conformational plasticity, arising from the shifting interplay between oligomerization and foldedness, reshapes how the same residue-level hydrophobic and electrostatic interactions can be differently deployed at the intermolecular scale, ultimately giving rise to distinct condensate material properties. We therefore hypothesize that evolution may have capitalized on these principles, selecting sequences with specific levels of conformational plasticity to endow condensates with functional properties suited to primordial or cellular needs. Such changes in the condensate microenvironment may, in turn, influence the structure of the same protein constituents or possibly other client proteins.

The case of disordered dimers is particularly intriguing, as it pushes the system to an extreme regime where stable dimers are formed between disordered peptides.^78^ At this edge of parameter space, we observe the emergence of a nematic phase, characterized by peptide alignment along a shared axis, with their charged C-termini oriented toward the RNA-enriched periphery (Figure 5A). The breaking of symmetry arises from the combination of extended peptide conformations and strong interpeptide interactions. This phenomenon recalls liquid–liquid crystalline phase separation seen in systems of anisotropic biopolymers, such as amyloid fibrils, where orientational ordering arises from rodlike interactions under crowded conditions.^79,80^ The emergence of an ordered phase from disordered components also resonates with prior work highlighting the role of transient ordered structures such as labile cross-β interactions in regulating disordered protein assembly,^81^ and bears some resemblance to the disorder-to-order transitions observed in more complex biological systems, where such transitions have been associated with condensate aging and pathological states.^82,83^ While the peptide model investigated here is highly simplified, reflecting its primordial origin, and lacks the compositional and sequence complexity of biological IDRs, which are typically longer and enriched in aromatic and polar residues,^84^ it nonetheless raises the possibility that similar physical principles may be at play. Specifically, aromatic and polar motifs may serve as molecular tethers for protein–protein interactions and, when coupled to extended conformations, could facilitate disorder-to-order transitions within condensates. Resonating with proposals that evolutionarily primitive RNA-binding proteins, lacking structured binding domains, may have relied on basic side chains interaction to engage RNA electrostatically on one side and backbone-mediated interaction with neighboring peptides on the other, to promote formation of protective granules.^85^

Together, our results illustrate how structural features such as foldedness and oligomerization shape the emergent properties of biomolecular condensates by balancing the intricate interplay of peptide–peptide and peptide–RNA interactions. Using a minimal, coarse-grained model grounded in experimental observations, we demonstrate that phase separation is not enabled solely by protein disorder but is also finely tuned by the degree of protein foldedness. From an evolutionary perspective, our results provide insights into how transitions from disordered to more ordered peptides, through a spectrum of intermediate states, may have shaped the material properties of early condensates, offering a functional context for the emergence of structural complexity. They also suggest that even subtle changes in structure can act as levers to tune condensate properties, providing a potential mechanism by which cells, or evolution, might regulate condensate material properties, without altering the sequence. More broadly, this work highlights the functional potential of the disorder–order continuum as a design space for biological regulation and points toward new directions for engineering synthetic condensates with programmable properties.

## Supplementary Material



## Notes

Coarse-grained modeling; phase diagrams for dimeric and monomeric systems; normalized phase diagrams for representative systems in the absence of RNA for dimeric and monomeric systems; anomalous diffusion exponent of the peptides extracted from the slope of the MSD on a log–log scale; log–log plot of the MSD curves of the peptides for each system; log–log plot of the RNA MSDs for each system; the relationship between foldedness and energetic and dynamic properties of the condensate; condensate density as a function of the frequency of intermolecular contacts; nematic order parameter in the presence or absence of RNA; time series of peptide–RNA interaction energies for all simulated systems; peptide–RNA averaged interaction energy plotted against peptide–peptide averaged interaction energy; examining the effect of the classification of monomeric and dimeric PA system on the conclusions regarding the condensate behavior (PDF)

## References

[1] Li P., Banjade S., Cheng H.-C., Kim S., Chen B., Guo L., Llaguno M., Hollingsworth J. V., King D. S., Banani S. F., Russo P. S., Jiang Q.-X., Nixon B. T., Rosen M. K. (2012). Phase Transitions
in the Assembly of Multivalent Signalling Proteins. Nature.

[2] Borcherds W., Bremer A., Borgia M. B., Mittag T. (2021). How Do Intrinsically
Disordered Protein Regions Encode a Driving Force for Liquid–Liquid
Phase Separation?. Curr. Opin. Struct. Biol..

[3] Hadarovich A., Singh H. R., Ghosh S., Scheremetjew M., Rostam N., Hyman A. A., Toth-Petroczy A. (2024). PICNIC Accurately
Predicts Condensate-Forming Proteins Regardless of Their Structural
Disorder across Organisms. Nat. Commun..

[4] Wang J., Choi J.-M., Holehouse A. S., Lee H. O., Zhang X., Jahnel M., Maharana S., Lemaitre R., Pozniakovsky A., Drechsel D., Poser I., Pappu R. V., Alberti S., Hyman A. A. (2018). A Molecular Grammar Governing the Driving Forces for
Phase Separation of Prion-like RNA Binding Proteins. Cell.

[5] Hazra M. K., Levy Y. (2021). Biophysics of Phase Separation of
Disordered Proteins Is Governed
by Balance between Short- And Long-Range Interactions. J. Phys. Chem. B.

[6] Biswas S., Potoyan D. A. (2025). Decoding Biomolecular
Condensate Dynamics: An Energy
Landscape Approach. PLOS Comput. Biol..

[7] Poudyal M., Patel K., Gadhe L., Sawner A. S., Kadu P., Datta D., Mukherjee S., Ray S., Navalkar A., Maiti S., Chatterjee D., Devi J., Bera R., Gahlot N., Joseph J., Padinhateeri R., Maji S. K. (2023). Intermolecular Interactions Underlie
Protein/Peptide
Phase Separation Irrespective of Sequence and Structure at Crowded
Milieu. Nat. Commun..

[8] Despotovic D., Tawfik D. S. (2021). Proto-proteins in
Protocells. ChemSystemsChem..

[9] Zhou H.-X., Nguemaha V., Mazarakos K., Qin S. (2018). Why Do Disordered and
Structured Proteins Behave Differently in Phase Separation?. Trends Biochem. Sci..

[10] Zhang Y., Li S., Gong X., Chen J. (2024). Toward Accurate Simulation of Coupling
between Protein Secondary Structure and Phase Separation. J. Am. Chem. Soc..

[11] Hernandez, G. ; Martins, M. L. ; Fernandes, N. P. ; Veloso, T. ; Lopes, J. ; Gomes, T. ; Cordeiro, T. N. Dynamic Ensembles of SARS-CoV-2 N-Protein Reveal Head-to-Head Coiled-Coil-Driven Oligomerization and Phase Separation. Nucleic Acids Research 2025, 53 (11), gkaf502, 10.1101/2024.12.02.626213. 40503686 PMC12159747

[12] Ramirez D. A., Hough L. E., Shirts M. R. (2024). Coiled-Coil
Domains Are Sufficient
to Drive Liquid-Liquid Phase Separation in Protein Models. Biophys. J..

[13] Conicella A. E., Dignon G. L., Zerze G. H., Schmidt H. B., D’Ordine A. M., Kim Y. C., Rohatgi R., Ayala Y. M., Mittal J., Fawzi N. L. (2020). TDP-43 α-Helical Structure
Tunes Liquid–Liquid
Phase Separation and Function. Proc. Natl. Acad.
Sci. U. S. A..

[14] Conicella A. E., Zerze G. H., Mittal J., Fawzi N. L. (2016). ALS Mutations Disrupt
Phase Separation Mediated by α-Helical Structure in the TDP-43
Low-Complexity C-Terminal Domain. Structure.

[15] Farag M., Borcherds W. M., Bremer A., Mittag T., Pappu R. V. (2023). Phase Separation
of Protein Mixtures Is Driven by the Interplay of Homotypic and Heterotypic
Interactions. Nat. Commun..

[16] Alshareedah I., Borcherds W. M., Cohen S. R., Singh A., Posey A. E., Farag M., Bremer A., Strout G. W., Tomares D. T., Pappu R. V., Mittag T., Banerjee P. R. (2024). Sequence-Specific
Interactions Determine Viscoelasticity and Ageing Dynamics of Protein
Condensates. Nat. Phys..

[17] Ginell G. M., Emenecker R. J., Lotthammer J. M., Keeley A. T., Plassmeyer S. P., Razo N., Usher E. T., Pelham J. F., Holehouse A. S. (2025). Sequence-Based
Prediction of Intermolecular Interactions Driven by Disordered Regions. Science.

[18] Cohen S. R., Banerjee P. R., Pappu R. V. (2024). Direct
Computations of Viscoelastic
Moduli of Biomolecular Condensates. J. Chem.
Phys..

[19] Cohen S. R., Banerjee P. R., Pappu R. V. (2024). Direct Computations of Viscoelastic
Moduli of Biomolecular Condensates. J. Chem.
Phys..

[20] Pal T., Wessén J., Das S., Chan H. S. (2024). Differential Effects
of Sequence-Local versus Nonlocal Charge Patterns on Phase Separation
and Conformational Dimensions of Polyampholytes as Model Intrinsically
Disordered Proteins. J. Phys. Chem. Lett..

[21] Tesei G., Lindorff-Larsen K. (2022). Improved Predictions of Phase Behaviour of Intrinsically
Disordered Proteins by Tuning the Interaction Range. Open Res. Eur..

[22] Welles R. M., Sojitra K. A., Garabedian M. V., Xia B., Wang W., Guan M., Regy R. M., Gallagher E. R., Hammer D. A., Mittal J., Good M. C. (2024). Determinants That
Enable Disordered Protein Assembly into Discrete Condensed Phases. Nat. Chem..

[23] Hazra M. K., Levy Y. (2020). Charge Pattern Affects
the Structure and Dynamics of Polyampholyte
Condensates. Phys. Chem. Chem. Phys..

[24] Chow C. F.
W., Lenz S., Scheremetjew M., Ghosh S., Richter D., Jegers C., Von Appen A., Alberti S., Toth-Petroczy A. (2025). SHARK-capture Identifies Functional Motifs in Intrinsically Disordered
Protein Regions. Protein Sci..

[25] Cubuk J., Alston J. J., Incicco J. J., Holehouse A. S., Hall K. B., Stuchell-Brereton M.
D., Soranno A. (2024). The Disordered
N-Terminal Tail of SARS-CoV-2 Nucleocapsid Protein Forms a Dynamic
Complex with RNA. Nucleic Acids Res..

[26] Kim J., Qin S., Zhou H.-X., Rosen M. K. (2024). Surface Charge Can Modulate Phase
Separation of Multidomain Proteins. J. Am. Chem.
Soc..

[27] Taneja I., Holehouse A. S. (2021). Folded Domain Charge Properties Influence the Conformational
Behavior of Disordered Tails. Curr. Res. Struct.
Biol..

[28] Ramirez D. A., Hough L. E., Shirts M. R. (2024). Coiled-Coil Domains Are Sufficient
to Drive Liquid-Liquid Phase Separation in Protein Models. Biophys. J..

[29] Jussupow A., Bartley D., Lapidus L. J., Feig M. (2025). COCOMO2: A
Coarse-Grained
Model for Interacting Folded and Disordered Proteins. J. Chem. Theory Comput..

[30] Hazra M. K., Levy Y. (2022). Affinity of Disordered
Protein Complexes Is Modulated by Entropy–Energy
Reinforcement. Proc. Natl. Acad. Sci. U. S.
A..

[31] Goldfarb D., Seal M., Edelstein I., Weil-Ktorza O., Metanis N., Levy Y., Longo L. (2024). RNA Binding and Coacervation
Promotes Preservation of Peptide Form and Function Across the Heterochiral-Homochiral
Divide. Chemistry.

[32] Seal M., Weil-Ktorza O., Despotović D., Tawfik D. S., Levy Y., Metanis N., Longo L. M., Goldfarb D. (2022). Peptide-RNA Coacervates
as a Cradle for the Evolution of Folded Domains. J. Am. Chem. Soc..

[33] Chen T., Chan H. S. (2015). Native Contact Density
and Nonnative Hydrophobic Effects
in the Folding of Bacterial Immunity Proteins. PLOS Comput. Biol..

[34] Sikosek T., Krobath H., Chan H. S. (2016). Theoretical
Insights into the Biophysics
of Protein Bi-Stability and Evolutionary Switches. PLOS Comput. Biol..

[35] Rogoulenko E., Levy Y. (2024). Skipping Events Impose
Repeated Binding Attempts: Profound Kinetic
Implications of Protein–DNA Conformational Changes. Nucleic Acids Res..

[36] Calinsky R., Levy Y. (2024). A pH-Dependent Coarse-Grained
Model for Disordered Proteins: Histidine
Interactions Modulate Conformational Ensembles. J. Phys. Chem. Lett..

[37] Joseph J. A., Reinhardt A., Aguirre A., Chew P. Y., Russell K. O., Espinosa J. R., Garaizar A., Collepardo-Guevara R. (2021). Physics-Driven
Coarse-Grained Model for Biomolecular Phase Separation with near-Quantitative
Accuracy. Nat. Comput. Sci..

[38] Noel J. K., Whitford P. C., Onuchic J. N. (2012). The Shadow
Map: A General Contact
Definition for Capturing the Dynamics of Biomolecular Folding and
Function. J. Phys. Chem. B.

[39] Allen, M. P. ; Tildesley, D. J. Computer Simulation of Liquids, 2nd ed.; Oxford University Press: Oxford, United Kingdom, 2017.

[40] Eastman P., Galvelis R., Peláez R. P., Abreu C. R. A., Farr S. E., Gallicchio E., Gorenko A., Henry M. M., Hu F., Huang J., Krämer A., Michel J., Mitchell J. A., Pande V. S., Rodrigues J. P., Rodriguez-Guerra J., Simmonett A. C., Singh S., Swails J., Turner P., Wang Y., Zhang I., Chodera J. D., De Fabritiis G., Markland T. E. (2024). OpenMM 8: Molecular Dynamics Simulation with Machine
Learning Potentials. J. Phys. Chem. B.

[41] Seal M., Edelstein I., Scolnik Y., Weil-Ktorza O., Metanis N., Levy Y., Longo L. M., Goldfarb D. (2025). RNA Binding and Coacervation Promote Preservation of Peptide Form and
Function across the Heterochiral–Homochiral Divide. Protein Sci..

[42] Sanchez-Burgos I., Herriott L., Collepardo-Guevara R., Espinosa J. R. (2023). Surfactants or Scaffolds?
RNAs of Varying Lengths Control the Thermodynamic Stability of Condensates
Differently. Biophys. J..

[43] Paloni M., Bussi G., Barducci A. (2021). Arginine Multivalency
Stabilizes
Protein/RNA Condensates. Protein Sci..

[44] Schrödinger, E. What Is Life? The Physical Aspect of the Living Cell ; with, Mind and Matter ; & Autobiographical Sketches; Cambridge University Press: Cambridge; New York, 1992.

[45] Hsu C. C., Buehler M. J., Tarakanova A. (2020). The Order-Disorder
Continuum: Linking
Predictions of Protein Structure and Disorder through Molecular Simulation. Sci. Rep..

[46] Majumdar A., Dogra P., Maity S., Mukhopadhyay S. (2019). Liquid–Liquid
Phase Separation Is Driven by Large-Scale Conformational Unwinding
and Fluctuations of Intrinsically Disordered Protein Molecules. J. Phys. Chem. Lett..

[47] Hazra M. K., Levy Y. (2021). Biophysics of Phase
Separation of Disordered Proteins Is Governed
by Balance between Short- And Long-Range Interactions. J. Phys. Chem. B.

[48] Shi S., Zhao L., Lu Z.-Y. (2024). Coarse-Grained
Modeling of Liquid–Liquid
Phase Separation in Cells: Challenges and Opportunities. J. Phys. Chem. Lett..

[49] Alshareedah I., Moosa M. M., Pham M., Potoyan D. A., Banerjee P. R. (2021). Programmable
Viscoelasticity in Protein-RNA Condensates with Disordered Sticker-Spacer
Polypeptides. Nat. Commun..

[50] Espinosa J. R., Joseph J. A., Sanchez-Burgos I., Garaizar A., Frenkel D., Collepardo-Guevara R. (2020). Liquid Network
Connectivity Regulates the Stability
and Composition of Biomolecular Condensates with Many Components. Proc. Natl. Acad. Sci. U. S. A..

[51] Holehouse A. S., Alberti S. (2025). Molecular Determinants
of Condensate Composition. Mol. Cell.

[52] Ahmed R., Liang M., Hudson R. P., Rangadurai A. K., Huang S. K., Forman-Kay J. D., Kay L. E. (2024). Atomic Resolution
Map of the Solvent Interactions Driving SOD1 Unfolding in CAPRIN1
Condensates. Proc. Natl. Acad. Sci. U. S. A..

[53] Koga S., Williams D. S., Perriman A. W., Mann S. (2011). Peptide–Nucleotide
Microdroplets as a Step towards a Membrane-Free Protocell Model. Nat. Chem..

[54] Patel C. K., Singh S., Saini B., Mukherjee T. K. (2022). Macromolecular
Crowding-Induced Unusual Liquid–Liquid Phase Separation of
Human Serum Albumin via Soft Protein–Protein Interactions. J. Phys. Chem. Lett..

[55] Zachrdla M., Savastano A., Ibáñez de Opakua A., Cima-Omori M.-S., Zweckstetter M. (2022). Contributions of the N-Terminal Intrinsically
Disordered Region of the Severe Acute Respiratory Syndrome Coronavirus
2 Nucleocapsid Protein to RNA-Induced Phase Separation. Protein Sci..

[56] Schwartz J. C., Wang X., Podell E. R., Cech T. R. (2013). RNA Seeds Higher-Order
Assembly of FUS Protein. Cell Rep..

[57] Hong Y., Najafi S., Casey T., Shea J.-E., Han S.-I., Hwang D. S. (2022). Hydrophobicity of
Arginine Leads to Reentrant Liquid-Liquid
Phase Separation Behaviors of Arginine-Rich Proteins. Nat. Commun..

[58] Li S., Chen J. (2024). Driving Forces of RNA Condensation Revealed through
Coarse-Grained
Modeling with Explicit Mg^2+^. Biophysics.

[59] Ramachandran V., Brown W., Gayvert C., Potoyan D. A. (2024). Nucleoprotein Phase-Separation
Affinities Revealed via Atomistic Simulations of Short Peptide and
RNA Fragments. J. Phys. Chem. Lett..

[60] Ramachandran V., Potoyan D. A. (2024). Atomistic Insights into the Reentrant Phase-Transitions
in Polyuracil and Polylysine Mixtures. J. Chem.
Phys..

[61] Maharana S., Wang J., Papadopoulos D. K., Richter D., Pozniakovsky A., Poser I., Bickle M., Rizk S., Guillén-Boixet J., Franzmann T. M., Jahnel M., Marrone L., Chang Y.-T., Sterneckert J., Tomancak P., Hyman A. A., Alberti S. (2018). RNA Buffers
the Phase Separation Behavior of Prion-like RNA Binding Proteins. Science.

[62] Langdon E. M., Qiu Y., Ghanbari
Niaki A., McLaughlin G. A., Weidmann C. A., Gerbich T. M., Smith J. A., Crutchley J. M., Termini C. M., Weeks K. M., Myong S., Gladfelter A. S. (2018). mRNA Structure
Determines Specificity of a polyQ-Driven Phase Separation. Science.

[63] Das S., Lin Y.-H., Vernon R. M., Forman-Kay J. D., Chan H. S. (2020). Comparative Roles of Charge, *π*, and Hydrophobic Interactions in Sequence-Dependent
Phase Separation
of Intrinsically Disordered Proteins. Proc.
Natl. Acad. Sci. U. S. A..

[64] Lin Y., Protter D. S. W., Rosen M. K., Parker R. (2015). Formation and Maturation
of Phase-Separated Liquid Droplets by RNA-Binding Proteins. Mol. Cell.

[65] Murthy A. C., Dignon G. L., Kan Y., Zerze G. H., Parekh S. H., Mittal J., Fawzi N. L. (2019). Molecular Interactions
Underlying
Liquid–liquid Phase Separation of the FUS Low-Complexity Domain. Nat. Struct. Mol. Biol..

[66] Ramšak M., Ramirez D. A., Hough L. E., Shirts M. R., Vidmar S., Eleršič
Filipič K., Anderluh G., Jerala R. (2023). Programmable
de Novo Designed Coiled Coil-Mediated Phase Separation in Mammalian
Cells. Nat. Commun..

[67] Czub M. P., Uliana F., Grubić T., Padeste C., Rosowski K. A., Lorenz C., Dufresne E. R., Menzel A., Vakonakis I., Gasser U., Steinmetz M. O. (2025). Phase Separation
of a Microtubule
Plus-End Tracking Protein into a Fluid Fractal Network. Nat. Commun..

[68] Martin E. W., Thomasen F. E., Milkovic N. M., Cuneo M. J., Grace C. R., Nourse A., Lindorff-Larsen K., Mittag T. (2021). Interplay of Folded
Domains and the Disordered Low-Complexity Domain in Mediating hnRNPA1
Phase Separation. Nucleic Acids Res..

[69] Daughdrill, G. W. Disorder for Dummies: Functional Mutagenesis of Transient Helical Segments in Disordered Proteins. In Intrinsically Disordered Proteins; Kragelund, B. B. , Skriver, K. , Eds.; Methods in Molecular Biology; Springer US: New York, NY, 2020; Vol. 2141, pp 3–20. 10.1007/978-1-0716-0524-0_1. 32696350

[70] Iwahara J. (2024). Transient
Helices with Functional Roles. Biophys. J..

[71] Wang S.-H., Zheng T., Fawzi N. L. (2024). Structure and Interactions of Prion-like
Domains in Transcription Factor Efg1 Phase Separation. Biophys. J..

[72] Mohanty P., Shenoy J., Rizuan A., Mercado-Ortiz J. F., Fawzi N. L., Mittal J. (2023). A Synergy between Site-Specific
and
Transient Interactions Drives the Phase Separation of a Disordered,
Low-Complexity Domain. Proc. Natl. Acad. Sci.
U. S. A..

[73] Haider R., Penumutchu S., Boyko S., Surewicz W. K. (2024). Phosphomimetic Substitutions
in TDP-43’s Transiently α-Helical Region Suppress Phase
Separation. Biophys. J..

[74] Wang A., Conicella A. E., Schmidt H. B., Martin E. W., Rhoads S. N., Reeb A. N., Nourse A., Ramirez Montero D., Ryan V. H., Rohatgi R., Shewmaker F., Naik M. T., Mittag T., Ayala Y. M., Fawzi N. L. (2018). A Single
N-terminal Phosphomimic Disrupts TDP-43 Polymerization, Phase Separation,
and RNA Splicing. EMBO J..

[75] Seim I., Posey A. E., Snead W. T., Stormo B. M., Klotsa D., Pappu R. V., Gladfelter A. S. (2022). Dilute
Phase Oligomerization Can
Oppose Phase Separation and Modulate Material Properties of a Ribonucleoprotein
Condensate. Proc. Natl. Acad. Sci. U. S. A..

[76] Banerjee P. R., Milin A. N., Moosa M. M., Onuchic P. L., Deniz A. A. (2017). Reentrant
Phase Transition Drives Dynamic Substructure Formation in Ribonucleoprotein
Droplets. Angew. Chem., Int. Ed..

[77] Lin Y.-H., Kim T. H., Das S., Pal T., Wessén J., Rangadurai A. K., Kay L. E., Forman-Kay J. D., Chan H. S. (2025). Electrostatics of
Salt-Dependent Reentrant Phase Behaviors
Highlights Diverse Roles of ATP in Biomolecular Condensates. eLife.

[78] Cubuk J., Incicco J. J., Hall K. B., Holehouse A. S., Stuchell-Brereton M. D., Soranno A. (2025). The Dimerization Domain of SARS-CoV-2
Nucleocapsid Protein Is Partially Disordered and Forms a Dynamic High-Affinity
Dimer. Cell Rep. Phys. Sci..

[79] Azzari P., Mezzenga R. (2023). Liquid-Liquid Crystalline
Phase Separation of Evolving
Amyloid Fibrils. Phys. Rev. Res..

[80] Fraccia T. P., Zanchetta G. (2021). Liquid–Liquid
Crystalline Phase Separation in
Biomolecular Solutions. Curr. Opin. Colloid
Interface Sci..

[81] Kato M., McKnight S. L. (2018). A Solid-State Conceptualization of
Information Transfer
from Gene to Message to Protein. Annu. Rev.
Biochem..

[82] Linsenmeier M., Hondele M., Grigolato F., Secchi E., Weis K., Arosio P. (2022). Dynamic Arrest and
Aging of Biomolecular Condensates
Are Modulated by Low-Complexity Domains, RNA and Biochemical Activity. Nat. Commun..

[83] Tejedor A. R., Collepardo-Guevara R., Ramírez J., Espinosa J. R. (2023). Time-Dependent Material
Properties of Aging Biomolecular Condensates from Different Viscoelasticity
Measurements in Molecular Dynamics Simulations. J. Phys. Chem. B.

[84] Martin E. W., Holehouse A. S., Peran I., Farag M., Incicco J. J., Bremer A., Grace C. R., Soranno A., Pappu R. V., Mittag T. (2020). Valence and
Patterning of Aromatic Residues Determine
the Phase Behavior of Prion-like Domains. Science.

[85] Kato M., Zhou X., McKnight S. L. (2022). How Do Protein Domains of Low Sequence
Complexity Work?. RNA.

