# Supplementary material for: How the Extent of Protein Folding and Oligomerization Modulate Condensate Formation and Properties
Source: J Phys Chem Lett. 2025 Oct 21;16(43):11248–58. doi: 10.1021/acs.jpclett.5c02083 (PMC12581155; doi:10.1021/acs.jpclett.5c02083)
Supplement: Supplementary file 1 [file jz5c02083_si_001.pdf]

Supplementary Information

Ilan Edelstein and Yaakov Levy

How the Extent of Protein Folding and Oligomerization Modulate Condensate Formation and Properties

Coarse-grained modeling

Our Cα implementation in OpenMM was inspired by Zhang et al.'s OpenABC software packages[1]. Bonds between adjacent beads were modeled using a harmonic bond potential of the form $V_b = k_b(r - r_0)^2$ with a force constant (k_b) of 20,000 kJ·mol⁻¹·nm⁻². Angular constraints were applied between three consecutive Cα beads using a harmonic angle potential of the form $V_\theta = k_\theta(\theta - \theta_0)^2$ with a force constant (k_θ) of 40 kJ·mol⁻¹·rad⁻². Soft torsion angles ($k_\phi(n = 1) = 1\ kJ\ mol^{-1}; k_\phi(n = 3) = 0.5\ kJ\ mol^{-1}$) were applied to 1-4 successive Cα beads using a potential of the form $V_\phi = k_\phi(1 + \cos(n\phi - \phi_0))$, where n is the periodicity. RNA nucleotides were modeled with three beads for the phosphate (P), sugar (S), and base (B). For RNA, angular constraints were applied to BSP, PSP, SPS, and BSS triplets[2] with a force constant (k_θ^{RNA}) of 83.68 kJ·mol⁻¹·rad⁻².

Interaction Type	Symbol	Force Constant (k)
Cα − Cα, RNA-RNA	k_b	20,000 kJ·mol⁻¹·nm⁻²
$\text{C}\alpha^{1-3}$	k_θ	40 kJ·mol⁻¹·rad⁻²
$\text{C}\alpha^{1-4}$	$k_\phi(n = 1)$	1 kJ·mol⁻¹
$\text{C}\alpha^{1-4}$	$k_\phi(n = 3)$	0.5 kJ·mol⁻¹
BSP, PSP, SPS, BSS	k_θ^{RNA}	83.68 kJ·mol⁻¹·rad⁻²

Table 1 *Force constants used in the coarse-grained model. Bonded interactions were applied between adjacent peptide and RNA beads, angles across three consecutive Cα beads, and dihedrals across four Cα beads. All values are expressed in kJ·mol⁻¹ with units corresponding to distance (nm) or angle (rad) as indicated. RNA angular constraints were applied to BSP, PSP, SPS, and BSS triplets.*

In order to implement a three-bead representation for RNA with Mpipi parametrization, short-range hydrophobic interactions were assigned to RNA base beads, long-range electrostatic to the phosphate beads and excluded volume interactions between sugar beads and all other beads. Hydrophobic pairwise parameterization between every Cα to B beads, charges, WF exponents, and cutoff values were defined according to the parametrization and definition of the original Mpipi model, and in a similar way as in the OpenABC package. Electrostatic interactions were modeled using the Debye-Hückel potential with an ionic strength of 100 mM (Debye length ≈

0.966 nm). The cutoff for electrostatic interactions was 3.5 nm, with a smoothing function ($O(\kappa)$) applied to avoid truncation artifacts. The potential used was of the form $V_{DH} = -k_{coul}\, B_k e^{-\frac{r}{\kappa}} (\epsilon_{water} r)^{-1} + O(\kappa)$ where ϵ_{water} is the dielectric constant of water, k_{coul} is the coulomb constant, B_k is the scaling factor depending on the Debye screening length (κ) and approaching 1 under the conditions that all the simulations were performed (a temperature of $300K$ and an ionic concentration of 100 mM). A smoothing function defined as $O(\kappa) = e^{-\frac{r_{cutoff}}{\kappa}} (\epsilon_{water} r_{cutoff})^{-1}$ was included to avoid truncation artifacts.

	**Electrostatic**	**Hydrophobic**
**Potential**	DH	WF
**Beads Type**	$C\alpha - C\alpha,\ C\alpha - P$	$C\alpha - C\alpha,\ C\alpha - B$

Table 2 *Nonbonded interaction potentials in the coarse-grained model. Electrostatic interactions were modeled using the Debye–Hückel (DH) potential between charged Cα and phosphate (P) beads. Hydrophobic interactions were modeled using the Wang–Frenkel (WF) potential between Cα–Cα and Cα–base (B) bead pairs.*

The structure of the PA peptide was encoded through specific intra- and intermolecular contacts pairs, extracted from a previously reported dimeric conformation[3]. The specific contact pairs (Fig S1 A-B), were identified via the shadow algorithm[4] (a light source is iteratively placed at the center of every atom, any neighboring atoms within a cutoff which have no shadow cast upon them are considered in contact), and modeled using a 12-10 Lennard-Jones potential of the form $V(r) = \epsilon_{inter/intra}(5\left(\frac{\sigma}{r}\right)^{12} - 6\left(\frac{\sigma}{r}\right)^{10})$. To gradually decrease the degree of foldedness, we tuned down the depth of the energy minima for the intramolecular contacts (ϵ_{intra}) (Fig. S1 B). Similarly, to decrease the degree of dimerization interactions, we tuned down the strength of the intermolecular contacts (ϵ_{inter}) (Fig. S1A).

Intermolecular contact pairs in all systems were applied to any pair of monomers present in the system. This was achieve by extending all intermolecular residue–residue pairs (from residue a in monomer$_i$ to residue b in monomer$_j$) such that all residues *a* from any monomer could interact with all corresponding residues *b* from all other monomers. This implementation allowed initially associated monomers that became separated to fully or partially re-oligomerize with any other available partner during the simulation. Overall, 15 combinations of ϵ_{intra} and ϵ_{inter} values we investigated, where ϵ_{intra} = 1, 2.5, or 4 and ϵ_{inter} = 0.001, 1, 2, 3, or 4.

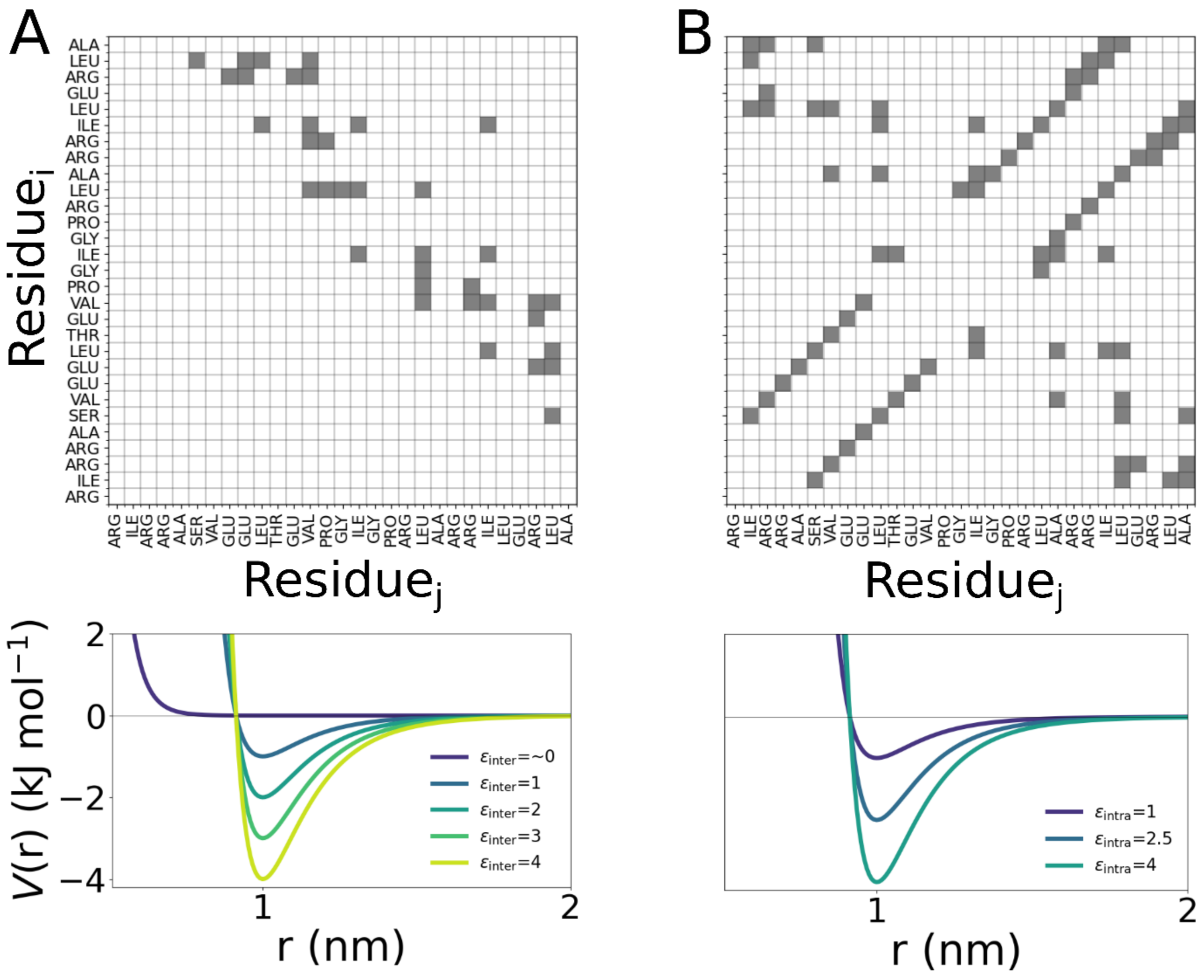

Figure S1. *Lennard-Jones (LJ) 12-10 potentials used to model specific inter- and intramolecular interactions. (**A**) Contact map of the specific intermolecular contacts pairs (i.e., the dimeric interface) defining the oligomerization interface between monomers (top). LJ potentials illustrating to the range of ϵ_{inter} values (0 to 4 kJ·mol^{-1}) used to tune oligomerization interface (bottom). (**B**) Contact map of the specific intramolecular contacts pairs (top). The axes show the sequence of the PA peptide. LJ potentials corresponding to the ϵ_{intra} values (1, 2.5, and 4 kJ·mol^{-1}) used to tune peptide foldedness (bottom). In both cases the real σ values depend on the specific i-j pairwise interaction; here, σ = 1 is used for illustration.*

Simulations were conducted using the OpenMM Middle Langevin Integrator with a friction coefficient of 1 ps^{-1} and the temperature of the heat bath set according to the simulation conditions. After 500 ns of equilibration phase to allow condensate formation as indicated by stable peptide-RNA interaction energies (Fig S11), three independent 1 μs runs were performed, yielding an aggregate simulation time of 3 μs per system.

Since in our model each peptide can partially or fully re-form an oligomerization interface depending on the strength of the ϵ_{inter} parameter, we first quantified the average number of oligomeric partners for each system. For each frame of every simulation, we computed the total pairwise interaction energy as the sum of all specific intermolecular contacts between every peptide i and peptide j. The resulting interaction energy matrices were then thresholded using a cutoff of –20 $kJ\ mol^{-1}$, such that two peptides were considered interacting if their pairwise energy fell below this value and non-interacting otherwise (Fig. S2A).

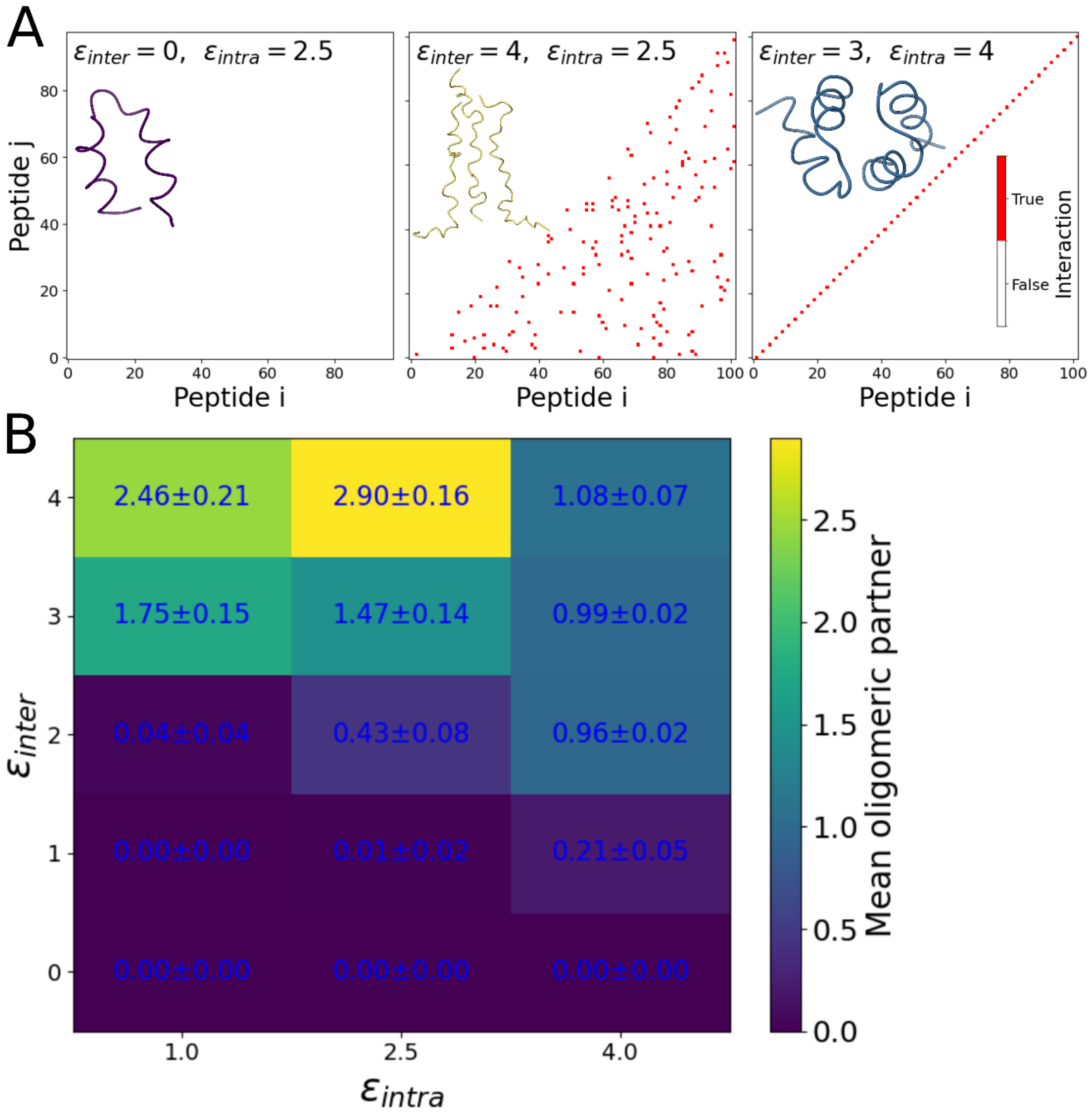

Figure S2. *(**A**) Representative intermolecular interaction energy maps, where two peptides were considered interacting if their pairwise energy was below* $-25\ kJ\ mol^{-1}$*, showing different pattern of interaction for folded monomers (left), disordered dimers (center) and folded dimers (right). The axes show the index number of the peptides participated in the simulations. (**B**) Average number of oligomeric partners for each system across all* $\epsilon_{inter} - \epsilon_{intra}$ *combinations. The three representative structures showed in (A) are colored according to the respective color scale computed in the heatmap.*

This cutoff value was chosen based on the assumption that systems at the extremes, monomers and folded dimers, should exhibit an average of 0 and 1 oligomeric partner, respectively. This assumption is further supported by the fraction of intermolecular contacts observed throughout the simulations (Fig. 1C). Using this criterion, we were able to count the number of peptides with which a given peptide interacted in each frame. These values were then averaged in a framewise manner. The averaged numbers of oligomeric partners and their standard deviations, reported in Fig. S2B, were computed from the aggregated vectors containing all frames across the replicates.

Phase diagrams were determined by clustering center of mass positions using DBSCAN in scikit-learn, on distance matrices precomputed under the minimal image convention. Dense phase volume was estimated using SciPy's Convex Hull algorithm. Critical temperature ($\boldsymbol{T_C}$) was obtained by fitting the density difference to the scaling law $(\Phi_D - \Phi_B)^{3.06} = d \cdot \left(1 - \frac{T}{T_c}\right)$, where Φ_D and Φ_B are the densities of the coexisting dense and dilute phase and d is a fitting parameter[5]. Systems were compared at different absolute temperaturewith phase diagrams normalized by their respective T_C, and analyzed at $T/T_C \approx 0.9$, a regime in which all systems exhibit phase separation (Fig. S2A–B).

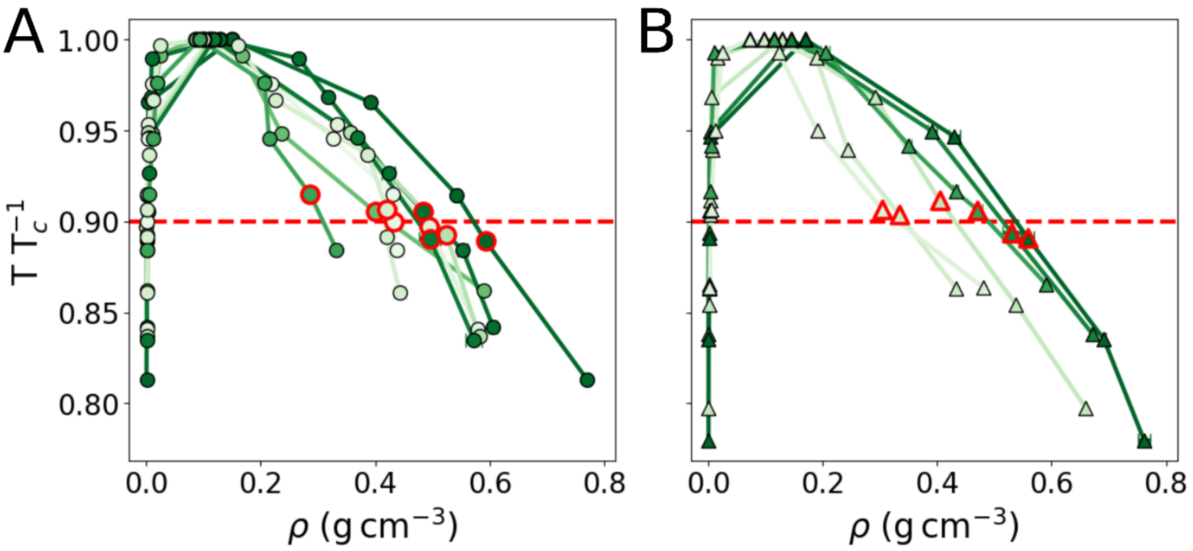

Figure S3. *(**A–B**) normalized phase diagrams for (**A**) dimeric and (**B**) monomeric systems, the red horizontal line indicates* $T/T_C = 0.9$*. The systems highlighted in red were chosen based on their proximity to* $T = 0.9\ T_c$ *and because they are all located in the two phase regime.*

Four representative PA systems were selected, one from each subtype group, and simulated in the absence of RNA as well as after mutating all arginine residues to lysines (R to K). The representative system for the ordered monomers group was $\epsilon_{intra} = 2.5,\ \epsilon_{inter} = 0$, for the disordered monomers group was $\epsilon_{intra} = 1,\ \epsilon_{inter} = 1$, for the ordered dimers group was $\epsilon_{intra} = 4,\ \epsilon_{inter} = 3$, and for the disordered dimers group was $\epsilon_{intra} = 2.5,\ \epsilon_{inter} = 4$.

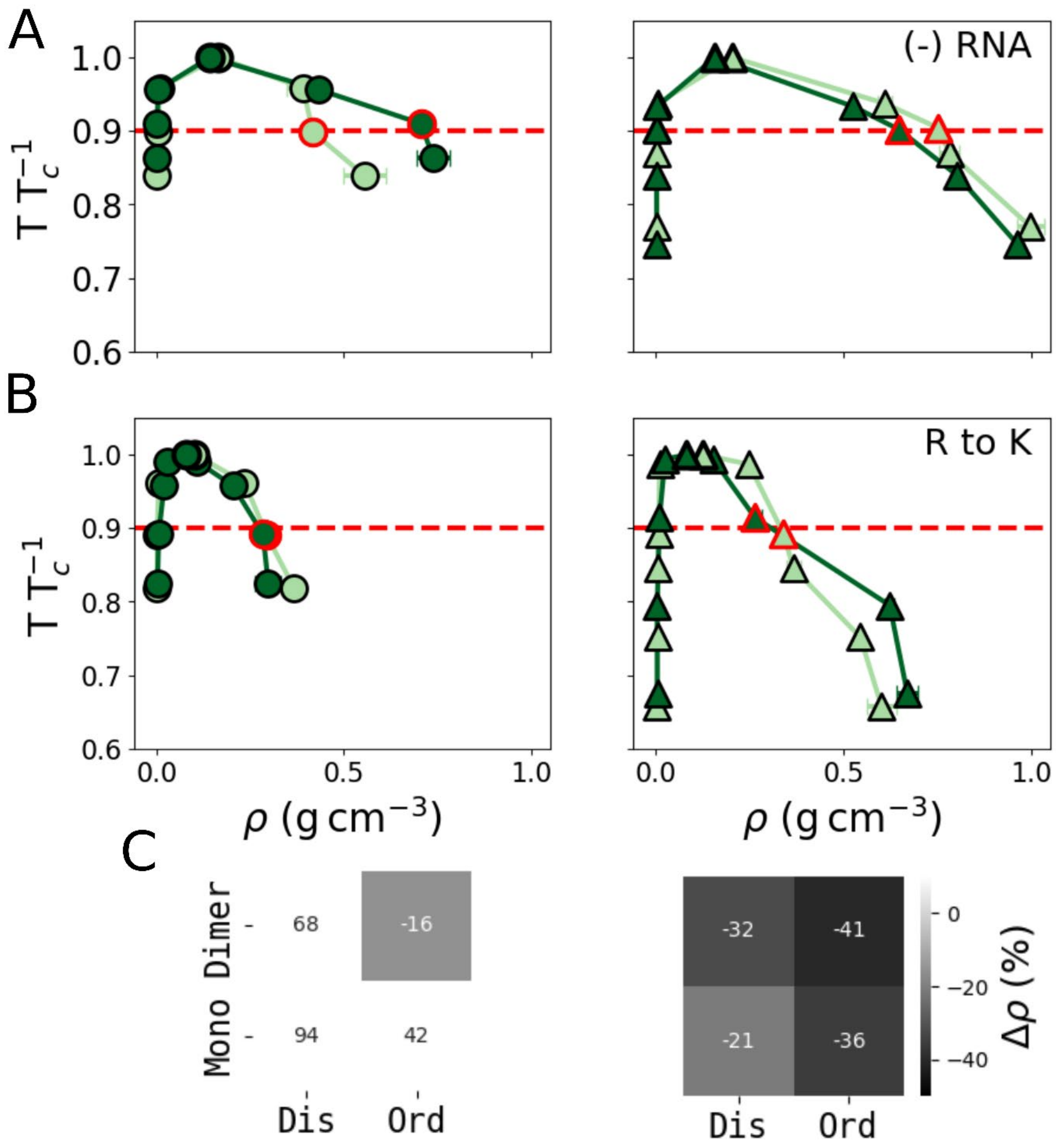

Figure S4. *(**A**) normalized phase diagrams for the representative systems in the absence of RNA for (left) dimeric and (right) monomeric systems, the red horizontal line indicates* $T/T_C = 0.9$*. The systems highlighted in red were chosen based on their proximity to the red line. (**B**) Same as in (A) but for the representative systems with the R to K substitution. (**C**) Relative change in condensate density from the systems in the presence of RNA to the systems upon RNA removal (left) and upon R to K substitutions (right).*

The diffusion coefficient (**D**) was determined from the slope of the mean squared displacement (MSD) of the center of mass positions of the peptide within the condensate. D was extracted by linear fitting of the MSD curve in its diffusive regime and divided by 6 (3D diffusion). Reported values represent the average D across all peptides within the condensate. To identify the fitting window and assess possible subdiffusive behavior, we also computed the diffusion exponent (**α**) defined as $\langle r(\tau)^2 \rangle = K_\alpha \tau^\alpha$, where K_α is the generalized diffusion coefficient and τ is the elapsed time. MSD curves were plotted on a log–log scale (Fig. S6), and α was obtained as the slope of the linear section of the MSD between τ =10 and 1000 ps (The window on which D was also extracted). The standard deviation for each system was computed by fitting the MSD of each peptide individually and calculating the error from the resulting distribution of values. All systems, apart from the four disordered dimer systems, exhibited α≈1, consistent with normal diffusion (Fig. S5A). In contrast, the disordered dimers displayed reduced α values (~0.8–0.83), indicating a slight subdiffusive regime.

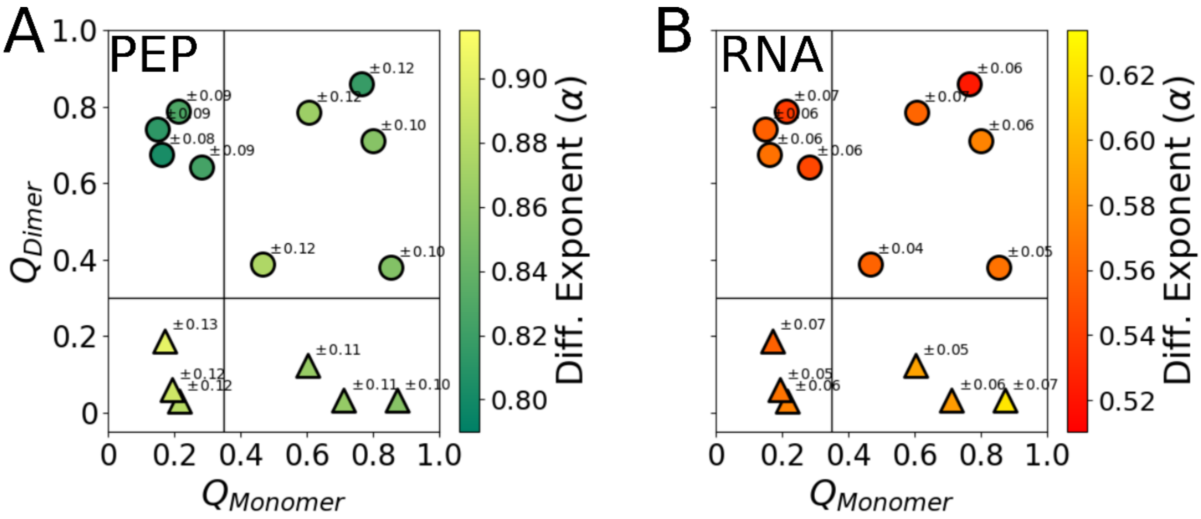

Figure S5. *(A) Anomalous diffusion exponent (α) of the peptides, extracted from the slope of the MSD on a log–log scale. The standard deviation is added for each system.* ***(B)*** *Same analysis as in (A), computed for the RNA molecules. Note that the color scale range differs between the peptide and RNA panels.*

Because the RNA molecules are much longer than the peptides, the MSD for each RNA was calculated at three distinct sites along its length. Specifically, MSD curves were computed at nucleotide positions 25, 50, and 75, corresponding to 25%, 50%, and 75% of the RNA length, respectively. This approach allowed us to assess whether diffusion at the periphery of the polymer differed from that at its center. Across all systems, the MSD curves on a log-log plot showed very similar trends (Fig S7), indicating comparable diffusive behavior along the RNA molecules. The RNA molecule consistently displayed subdiffusive behaviour in all system (fitting window between τ =10 and 1000 ps), with α values ranging between ~0.5–0.6 (Fig. S5B). The mean values reported in Fig. S5B were averaged over the three positions, since their

values were found to be close to one another (Fig. S7). The standard deviation was computed by independently fitting the three position of each RNA molecule.

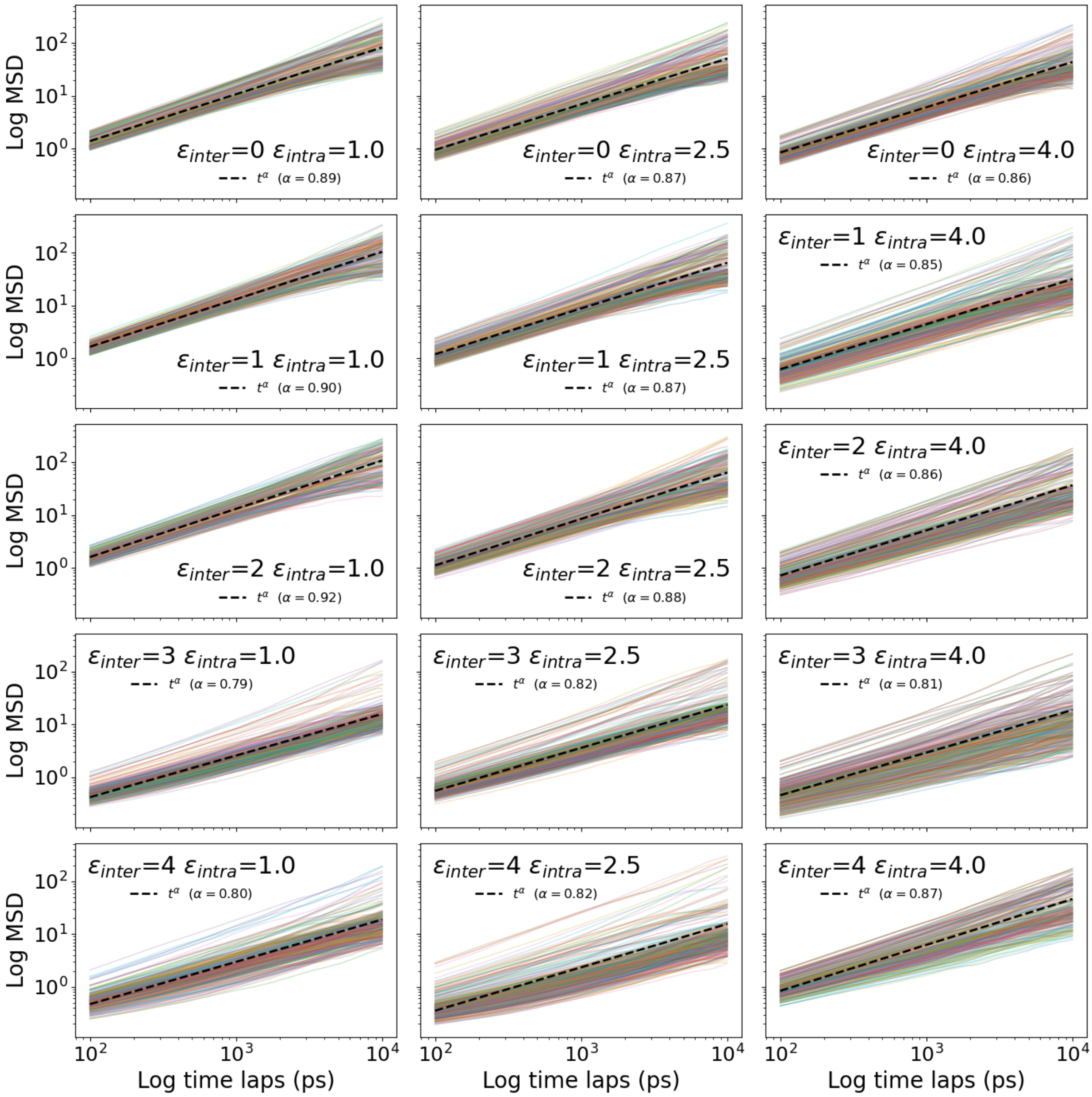

Figure S6. *Log-log plot of the MSD curves of the peptides for each system. Columns correspond to increasing values of* ϵ_{intra}*(left to right), and rows to increasing values of* ϵ_{inter} *(top to bottom). Individual peptide MSD curves are shown in color, and the fitted average across peptides is shown as a black dashed line.*

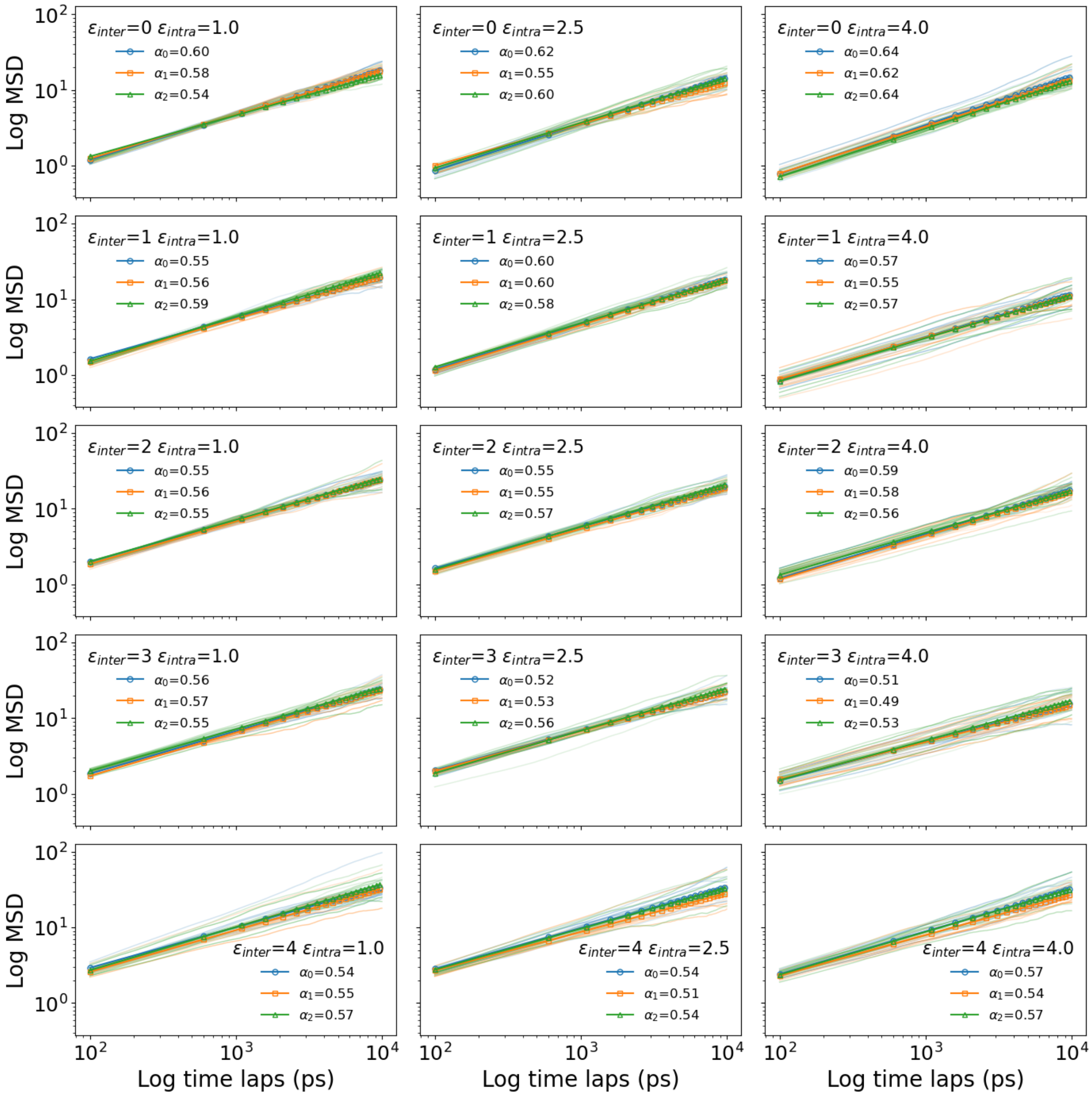

Figure S7. *Log-log plot of the RNA MSDs for each system. Columns correspond to increasing values of* ϵ_{intra}*(left to right), and rows to increasing values of* ϵ_{inter} *(top to bottom). The blue, orange and green linear fit represent the three sites from which MSD was compute for each RNA molecule.* α_0 *is the fitted average diffusion exponent of nucleotide 25,* α_1 *of nucleotide 50 and* α_2 *of nucleotide 75.*

Foldedness was defined as a measure of average ‘helicity’ of the peptide located in the condensate. Helicity was computed using MDAnalysis HELANAL module that quantifies the geometry of helices in proteins based solely on Cα atoms. In this context, increase in helicity refers to reduction in average helical twist angle computed for the helical regions and vice versa. To construct a normalized foldedness scale, the average helical twist angle for each system was first rescaled between 0 and 1, then inverted (1 minus the normalized value) so that lower twist angles (more helical structure retained) yield higher foldedness scores, and higher twist angles (broken helices) yield lower foldedness scores. In parallel, $\boldsymbol{Q_{Monomer}}$ and $\boldsymbol{Q_{Dimer}}$, defined as

the fraction of intramolecular or intermolecular native contacts, respectively, maintained during the simulation. A native contact was considered formed if the corresponding pairwise residue distance was within 1.1σ. These values were then normalized by the total number of possible contacts per monomer or dimer, yielding a score between 0 and 1. As expected, $Q_{Monomer}$ correlates strongly to the foldedness scale (Fig. S8B).

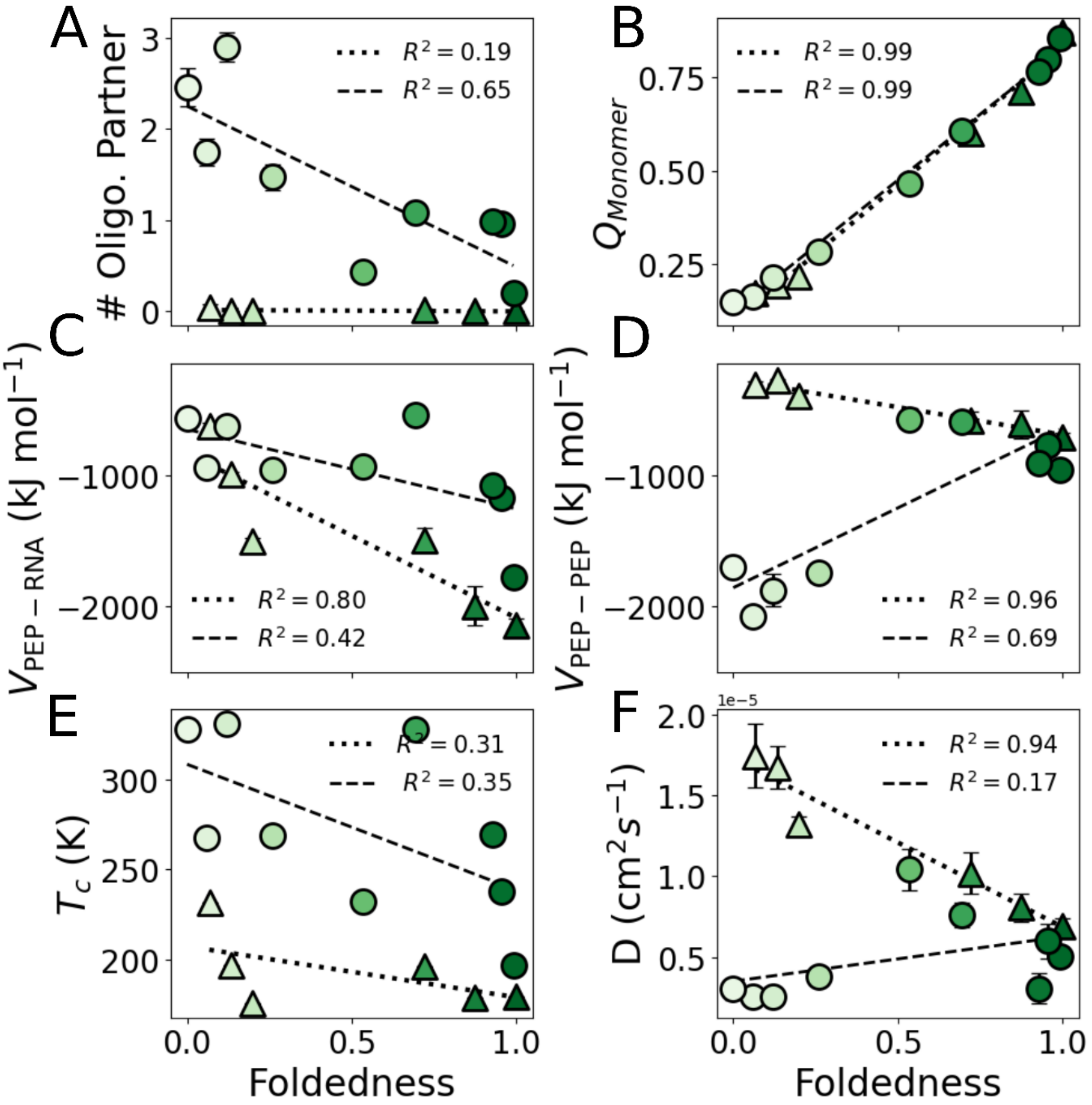

Figure S8. *The relationship between foldedness and energetic and dynamic properties of the condensate. (**A**) Average number of oligomeric partner as function of the foldedness scale (**B**) Foldedness strongly correlates with the intramolecular fraction of contacts* $Q_{Monomer}$. *(**C**) Peptide-RNA interaction energy negatively correlates with foldedness. (**D**) Peptide-peptide interaction energy differently correlates with foldedness. (**E**) Foldedness weakly correlates with* T_c. *(**F**) Diffusivity strongly negatively correlates with foldedness for the monomeric system and more weakly positively correlates for dimeric systems.*

The radius of gyration ($\boldsymbol{R_g}$) of peptides was computed using MDTraj[6]. To estimate the structural changes upon entering the condensate, R_g and helicity values for the bulk phase were computed and averaged from three independent simulations performed on a single PA dimer, in the exact same conditions as the corresponding parent system. Notably, disordered dimers exhibited the largest increase in R_g upon entering the condensate, indicating expansion upon phase separation (Fig. S9D).

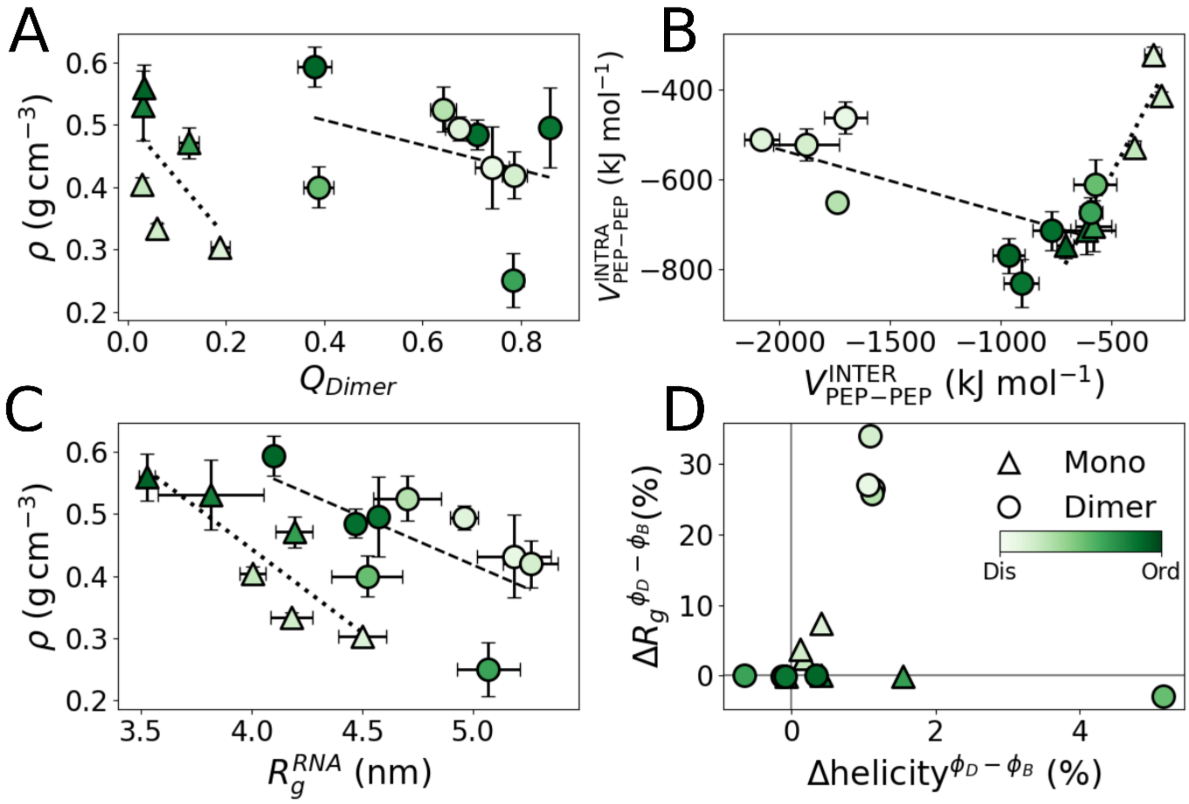

Figure S9. *(**A**) Condensate density as a function of the frequency of intermolecular contacts. (**B**) Intramolecular interactions within the peptides as a function of the intermolecular interactions energy between the peptides (in each case sum of the electrostatic and hydrophobic contributions). (**C**) Condensate density negatively correlates with RNA radius of gyration. Foldedness is colored according to the degree of folding of the peptides constituents. (**D**) Relative change in average radius of gyration and helicity of the peptides constituents upon entering the condensate.* ϕ_D *refers to the values in the dense phase and* ϕ_B *in the bulk phase, respectively.*

The **nematic order** describes the tendency of the system to order itself toward a common axis with a value between 0 and 1. For a system of N peptides with orientation unit vectors e, Q the ordering matrix was defined as $Q = \frac{1}{2N}\sum_{i=1}^{N} 3e_{\alpha}^{i}\, e_{\beta}^{i} - \delta_{\alpha\beta}$ where $\alpha, \beta \in \{x, y, z\}$. Diagonalization of Q yields its eigenvalues, and the nematic order parameter S was then defined as the largest eigenvalue of Q.

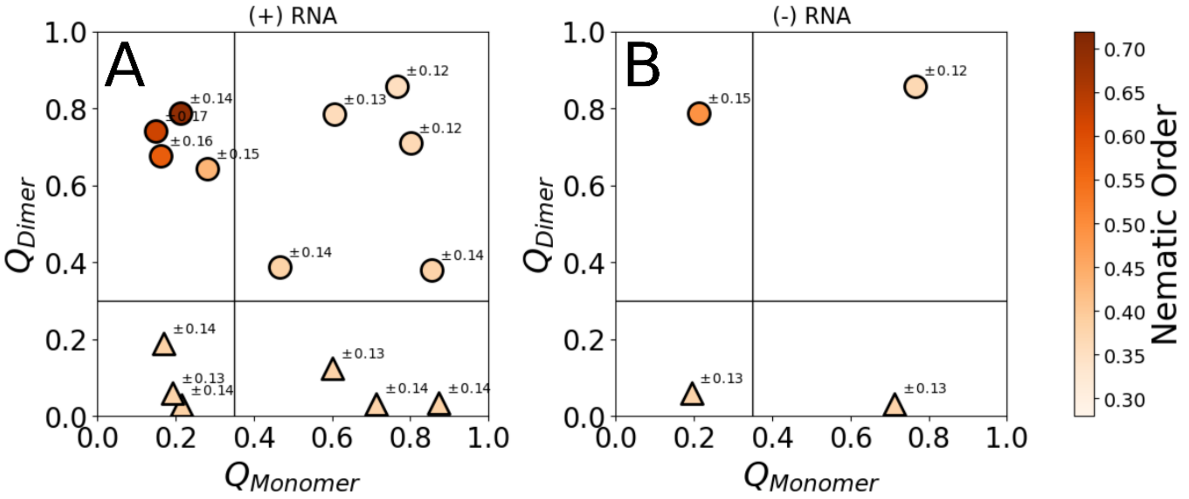

Figure S10. *Nematic order parameter (A) In the presence of RNA, (**B**) In the absence of RNA.*

Interaction energies within the dense phase were computed for each frame as the sum of electrostatic and hydrophobic interactions, both between peptides and between peptides and RNA. For each system, replicate means were calculated, and the overall mean and standard deviation were obtained across replicates. $V_{PEP-PEP}$ includes only the interaction energy between peptides, or in other words the energy of peptide or RNA interaction with themselves is not included.

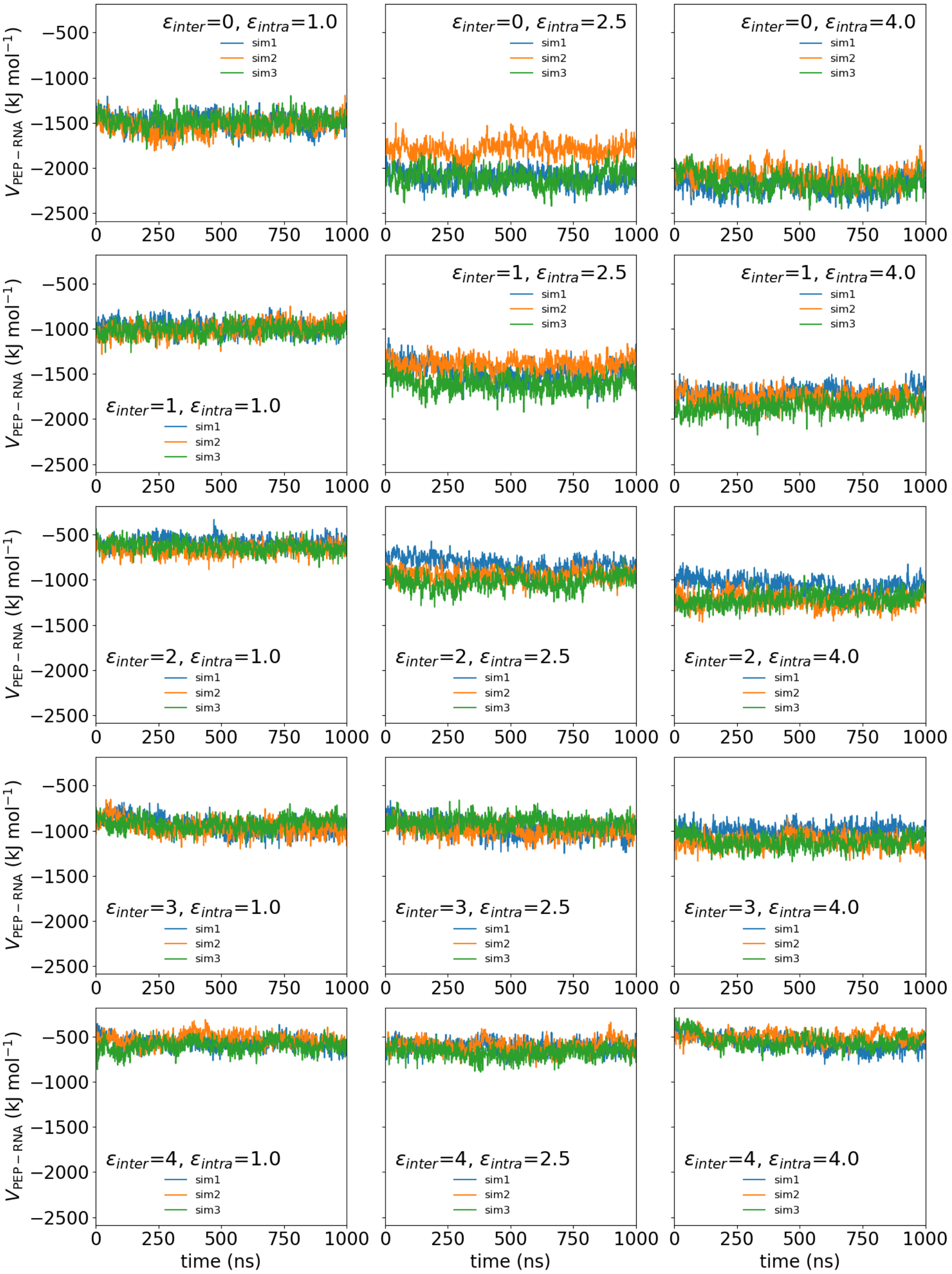

Figure S11. *Time series of peptide–RNA interaction energies for all simulated systems. Each subplot shows the distribution of interaction energies across three independent 1 µs simulations. Columns correspond to increasing values of ϵ_{intra}(left to right), and rows to increasing values of ϵ_{inter} (top to bottom). The consistent and monotonic behavior across all systems suggest that equilibration was achieved.*

To clarify the apparently opposing role of RNA in condensate stability, we performed simulations of each representative group with RNA molecules of shorter and longer lengths, while keeping the system temperature constant (i.e., the simulations with altered RNA length were ran at the same temperature as the corresponding parent system with 100 nucleotides). Under these isothermal conditions, we observed that increasing RNA length consistently enhanced the average peptide–peptide interaction energy across all groups (Fig. S12). Notably, systems with preferred monomeric oligomeric states exhibited lower baseline peptide–peptide interactions, as reflected by the rightward shift in their average peptide-peptide interaction energy (Fig. S12).

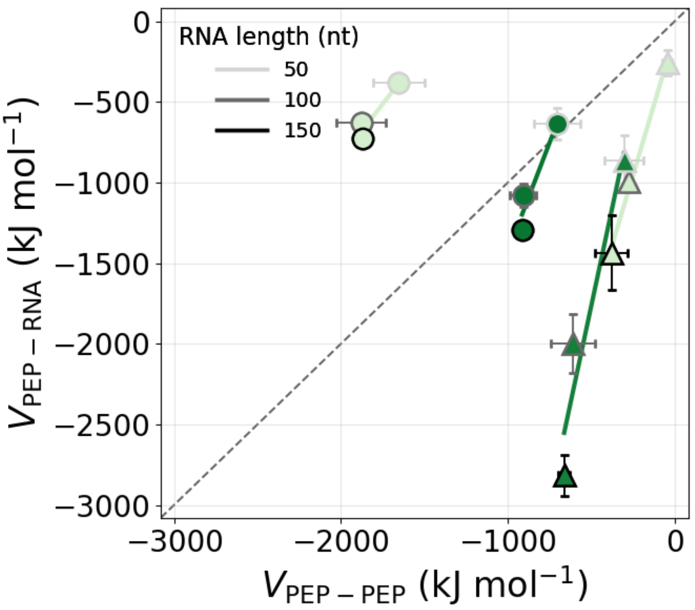

Figure S12. *Peptide–RNA averaged interaction energy plotted against peptide–peptide averaged interaction energy for each PA variant subgroup. Marker edge color indicates the length of the four RNA molecules present in the simulations: light grey = 50 nucleotides, dark grey = 100 nucleotides (same as in Fig. 3), and black = 150 nucleotides.*

To ensure that our conclusions are not biased by methodological choices, we performed two control analyses. First, we evaluated whether the distinct behavior of the disordered dimer group warrants its exclusion from the correlation analyses. Second, we tested the robustness of our classification threshold between monomers and dimers to assess whether shifting the boundary affects the overall trends.

Disordered dimers exhibit the most divergent behavior (Fig. 5A), raising the possibility that their inclusion could disproportionately influence observed correlations, particularly the anticorrelation along the oligomerization axis between stability (T_c) and peptide–RNA interaction energies, and between diffusivity and peptide–peptide interaction energies. However, excluding this group did not alter the overall trends (Fig. S13). While the directionality of some correlations within the dimeric system (e.g., between diffusivity and peptide–RNA interactions, or Tc and peptide–peptide interactions) was somewhat affected, our main conclusions do not rely on these particular correlations.

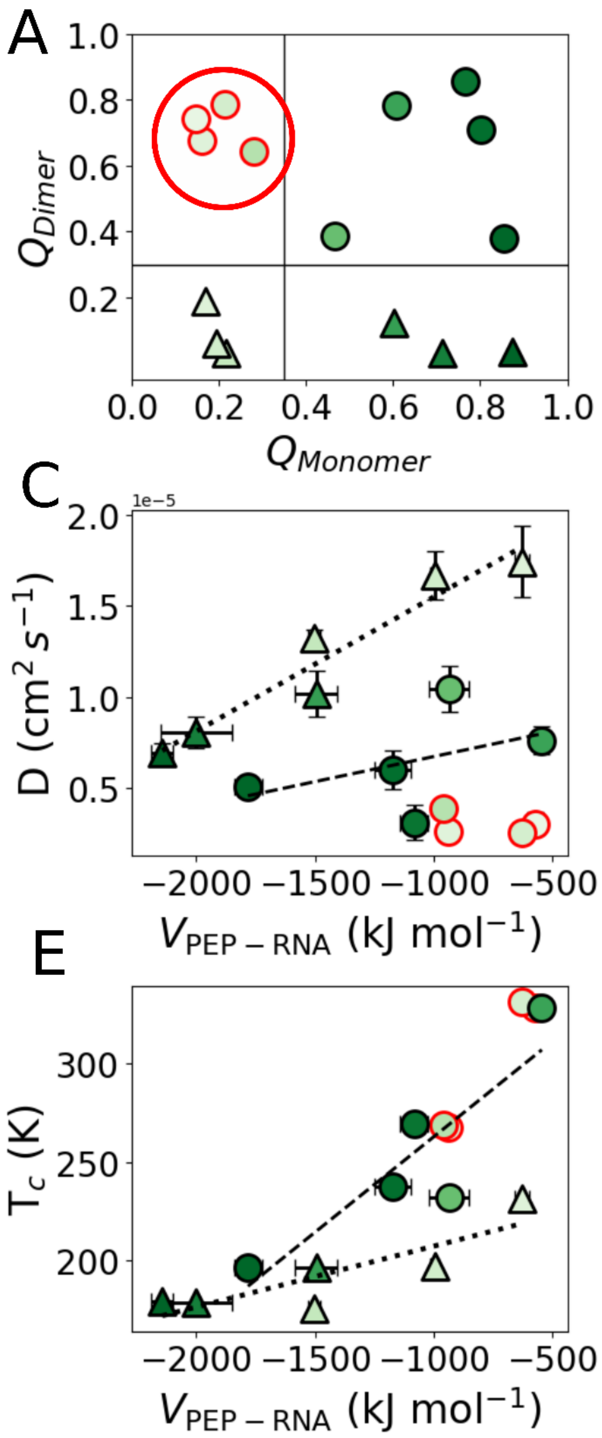

Figure S13. *The Dimer^{Dis} (highlighted in red) are excluded from the correlations (**A**) Classification of the 15 conformational variants. (**B**) Condensate averaged density as a function of the foldedness of the peptide constituents. (**C**) Average diffusion coefficient as a function of the mean interaction energy between peptides and RNA (**D**) and between peptides.(**E**) T_c as a function of the mean interaction energy between peptides and RNA (**F**) and between peptides.*

Next, we increased the threshold for dimer classification from 0.3 to 0.5. As expected this resulted in the reassignment of the two intermediate systems from dimers to monomers (Fig. 14A). This reclassification did not alter the correlations (Fig. 4B-F). We therefore conclude that similar conclusions would be reached even if the two intermediate systems were labeled as monomers.

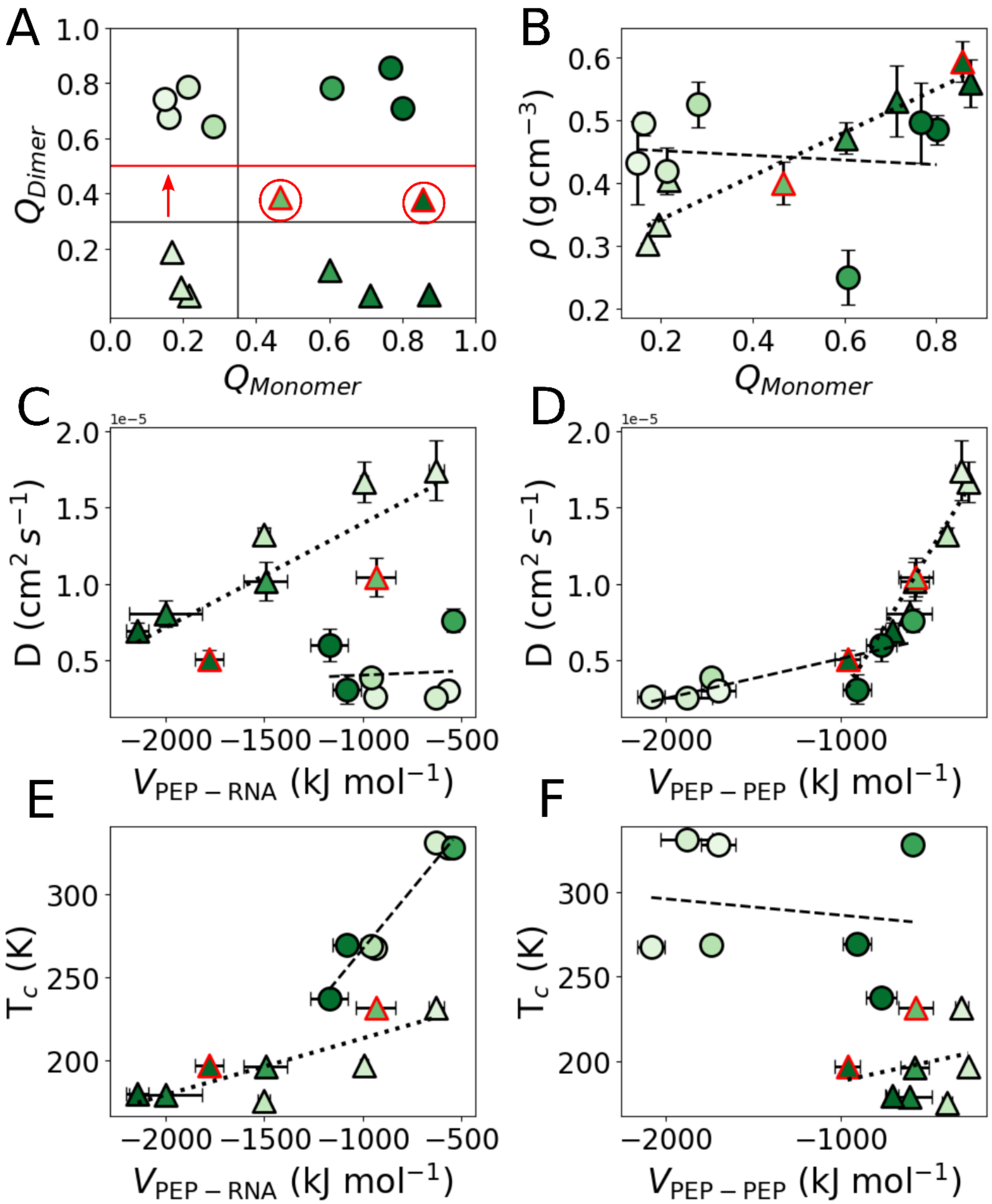

Figure S14. *Examining the effect of the classification of monomeric and dimeric PA system on the conclusions regarding the condensate behavior. The threshold for a system being classified as Dimeric is increased to* $Q_{Dimer} > 0.5$*; The 2 systems switching from a dimer to a monomer classification are highlighted in red. (**A**) Classification of the 15 conformational variants. (**B**) Condensate averaged density as a function of the foldedness of the peptide constituents. (**C**) Average diffusion coefficient as a function of the mean interaction energy between peptides and RNA (**D**) and between peptides.(**E**)* T_c *as a function of the mean interaction energy between peptides and RNA (**F**) and between peptides.*

References

(1) Liu, S.; Wang, C.; Latham, A. P.; Ding, X.; Zhang, B. OpenABC Enables Flexible, Simplified, and Efficient GPU Accelerated Simulations of Biomolecular Condensates. *PLOS Comput. Biol.* **2023**, *19* (9), e1011442. https://doi.org/10.1371/journal.pcbi.1011442.

(2) Pal, A.; Levy, Y. Structure, Stability and Specificity of the Binding of ssDNA and ssRNA with Proteins. *PLoS Comput. Biol.* **2019**, *15* (4), e1006768. https://doi.org/10.1371/journal.pcbi.1006768.

(3) Seal, M.; Weil-Ktorza, O.; Despotović, D.; Tawfik, D. S.; Levy, Y.; Metanis, N.; Longo, L. M.; Goldfarb, D. Peptide-RNA Coacervates as a Cradle for the Evolution of Folded Domains. *J. Am. Chem. Soc.* **2022**, *144* (31), 14150–14160. https://doi.org/10.1021/jacs.2c03819.

(4) Noel, J. K.; Whitford, P. C.; Onuchic, J. N. The Shadow Map: A General Contact Definition for Capturing the Dynamics of Biomolecular Folding and Function. *J. Phys. Chem. B* **2012**, *116* (29), 8692–8702. https://doi.org/10.1021/jp300852d.

(5) Espinosa, J. R.; Garaizar, A.; Vega, C.; Frenkel, D.; Collepardo-Guevara, R. Breakdown of the Law of Rectilinear Diameter and Related Surprises in the Liquid-Vapor Coexistence in Systems of Patchy Particles. *J. Chem. Phys.* **2019**, *150* (22), 224510. https://doi.org/10.1063/1.5098551.

(6) McGibbon, R. T.; Beauchamp, K. A.; Harrigan, M. P.; Klein, C.; Swails, J. M.; Hernández, C. X.; Schwantes, C. R.; Wang, L.-P.; Lane, T. J.; Pande, V. S. MDTraj: A Modern Open Library for the Analysis of Molecular Dynamics Trajectories. *Biophys. J.* **2015**, *109* (8), 1528–1532. https://doi.org/10.1016/j.bpj.2015.08.015.
